# Epidemiology, outcomes of treatment, and survival of cervical cancer in Kazakhstan: a nationwide population-based study

**DOI:** 10.3389/fonc.2026.1820759

**Published:** 2026-06-05

**Authors:** Aidana Rakhmankulova, Yesbolat Sakko, Abduzhappar Gaipov, Kuralay Atageldiyeva, Talshyn Ukybassova, Assem Suleimenova, Yerbolat Iztleuov, Gulzhanat Aimagambetova

**Affiliations:** 1 Department of Biomedical Sciences, School of Medicine, Nazarbayev University, Astana, Kazakhstan; 2 Department of Medicine, School of Medicine, Nazarbayev University, Astana, Kazakhstan; 3 Clinical Academic Department of Women’s Health, CF “University Medical Center”, Astana, Kazakhstan; 4 Kazakh National Institute of Oncology and Radiology, Almaty, Kazakhstan; 5 Department of Radiology, Marat Ospanov West-Kazakhstan Medical University, Aktobe, Kazakhstan; 6 Department of Surgery, School of Medicine, Nazarbayev University, Astana, Kazakhstan

**Keywords:** ASIR, ASMR, cervical cancer, DALY, forecast, Kazakhstan, survival, vaccination

## Abstract

**Background:**

Cervical cancer remains one of the major medical and public health issues in Kazakhstan. In 2024, Kazakhstan launched a campaign to vaccinate 11- to 13-year-old girls, demonstrating successful implementation. This is a retrospective, population-based study aiming to investigate epidemiology, treatment outcomes, and survival of the cervical cancer pre-vaccination trends in a 10-year period (2014–2023).

**Methods:**

Data were obtained from the Electronic Registry of Oncological Patients, which records clinically relevant encounters (inpatient admissions, outpatient visits, and official follow-up).

**Results:**

Cervical cancer prevalence increased gradually over a 10-year period, while age-standardized incidence rate (ASIR) and age-standardized mortality rate (ASMR) peaked at approximately 2022 (17.22 and 6.92 per 100,000 women, respectively). Disability-adjusted life year (DALY) rates increased sharply to 255.24 per 100,000 women in 2022, and 5-year survival was 65.3%. Despite a moderate 5-year overall survival, outcomes in advanced stages are poor and contribute disproportionately to years of life lost. Chemotherapy, radiation, and later stages showed an increased adjusted hazard ratio, and more days lost were seen from the restricted mean survival time (RMST) analysis.

**Conclusion:**

This nationwide, registry-based analysis from Kazakhstan reveals that cervical cancer remains a major and growing public health burden. This study’s findings suggest a baseline cervical cancer epidemiology for later comparison once the human papillomavirus vaccination program is fully implemented with high coverage.

## 1 Introduction

Cervical cancer is a malignant condition affecting women worldwide and remains a global public health problem (1). According to the World Health Organization (WHO) global data, in 2022, there were approximately 662,000 new cervical cancer cases and 349,000 deaths worldwide (2). In 2020, cervical cancer incidence ranged from 2.2 per 100,000 women in Iraq to 84.6 per 100,000 women in Eswatini (1). Because of unequal access to cervical cancer screening and treatment, the vast majority of cervical cancer deaths (91%) occur in low- and middle-income countries (LMICs) (1, 3). Considering that cervical cancer is one of the preventable malignancies, the WHO proposed and is implementing the global Cervical Cancer Elimination Initiative (4, 5). The strategy aims to eliminate cervical cancer as a public health problem, defined as reaching and maintaining an incidence<4 cases per 100,000 women-year (6).

In Kazakhstan, cervical cancer incidence has remained high for the past two decades. According to the data available for 2018, the crude rate of cervical cancer incidence in Kazakhstan was as high as 18.6 per 100,000 women. Moreover, the incidence increased in a period of 10 years (2009–2018) from 16.3 to 19.5 (7, 8). The association of cervical cancer development with high-risk types of human papillomavirus (HPV) infection was established in the 1990s (9). Since that time, the prevalence of high-risk HPV infection in Kazakhstan has been well-investigated by local researchers (10–16). The researchers reported that the prevalence of HPV-16 and HPV-18 types among the female and male populations in Kazakhstan was approximately 26% in 2016 (15). Later studies identified the prevalence of high-risk HPV types among women attending gynecological clinics ranging between 39% and 43% (10, 12, 16). In a further study, researchers found a high prevalence of HPV infection among women with abnormal cervical cytology—62.4% were positive for high-risk HPV (13). Thus, the high prevalence of high-risk HPV types along with the absence of a country-wide organized HPV vaccination program before 2024 (17, 18) contributed to the growing incidence of cervical cancer in Kazakhstan (7, 8). The first HPV vaccination campaign in Kazakhstan started in 2013 but was discontinued in 2015 because of misinformation and lack of awareness both in the general population and among healthcare workers. A second, better-prepared national program was launched in 2024 and is in progress now, targeting 11- to 13-year-old girls voluntarily (19).

Another contributing factor to the cervical cancer incidence growth is the low coverage of the cervical cancer screening program in Kazakhstan. While the cervical screening program was well-developed and implemented more than a decade ago (10), multiple factors affect the screening attendance (20); thus, the screening coverage remains low and inconsistent over recent years, with the lowest coverage of 46% in 2016 and 52% in 2023 and the highest coverage in 2021–2022 (92%) (21, 22).

Therefore, considering the high prevalence of HPV infection, inconsistent screening coverage, the absence of a nationwide HPV vaccination program before 2024, and increasing trends in the cervical cancer incidence, this study aimed to investigate the overall epidemiology and treatment outcomes of cervical cancer in Kazakhstan for the past 10 years (2014–2023) using the National Electronic Healthcare System.

## 2 Methods

### 2.1 Study design and population

This is a retrospective, population-based study analyzing the data from the National Electronic Registry of Oncological Patients (EROP). Cervical cancer cases were identified using ICD-10 codes C53, C53.0, C53.1, C53.8, and C53.9. The dataset includes all clinically relevant records (inpatient admissions, outpatient visits, and official follow-up) across participating public healthcare facilities (23). All recorded individuals have a unique, lifelong population registry number (RpnID), providing safety for their personal data. Missing data were also searched through UNEHS’s Electronic Registry of Inpatients and Outpatients, a national digital database launched in late 2013 to consolidate inpatient medical records across Kazakhstan’s healthcare institutions, using RpnIDs (24). EROP has several forms, each of which is responsible for different data collection (23). The data for population number in calculating rates was obtained from the Statistics Committee under the Ministry of National Economy of the Republic of Kazakhstan (25).

### 2.2 Exposures and covariates

Individual data included RpnID, demographic, and clinical characteristics. Grouping of data was performed based on ethnic origin, age, and body mass index (BMI) groups. There were more than 100 ethnicities reported, which were grouped into 3 major groups: Kazakh, Russian, and others. Age groups were divided by 5 years (adapted from the Centers for Disease Control and Prevention for epidemiological analysis), with the exception of the 0–14 group, as the disease is more frequently found in older ages than in children. BMI was calculated for those with data available and grouped into internationally accepted groups (underweight, healthy weight, overweight, obesity, and severe obesity).

### 2.3 Tumor staging/characteristics

Tumor staging was shown in 2 classification systems [International Federation of Gynecology and Obstetrics (FIGO) and TNM]. Staging was done based on the Kazakhstani national guideline (26) that aligned with the American Joint Committee on Cancer (AJCC) TNM classification and the FIGO staging system (27).

Stage migration was not anticipated, given the uniform use of the TNM-based classification system throughout. For analysis, cancer stages were grouped into larger ones (I, II, III, and IV) due to a lack of precise diagnosis written in the database. Histological types of tumors were grouped into 3 categories. All the missing data were reported as it is and shown in all tables under the group “missing”.

### 2.4 Outcome assessment

Age-standardized incidence, mortality, and prevalence rates per 100,000 women were calculated using direct age standardization. Age-specific rates were multiplied by the corresponding WHO standard population weights and summed across age groups. Prevalence was defined as the number of women alive with cancer on 31 December of each calendar year. Exact formulas are shown below (Equations 1–3):

Age-standardized incidence rate (ASIR) per 100,000 women.

(1) $${\text{ASIR}}_{t}=\sum_{i}\left(\frac{{\text{Cases}}_{it}}{{\text{Population}}_{it}}\times 100,000\times w_{i}\right)$$

Age-standardized mortality rate (ASMR) per 100,000 women.

(2) $${\text{ASMR}}_{t}=\sum_{i}\left(\frac{{\text{Deaths}}_{it}}{{\text{Population}}_{it}}\times 100,000\times w_{i}\right)$$

Age-standardized prevalence per 100,000 women.

(3) $${\text{ASP}}_{t}=\sum_{i}\left(\frac{{\text{Prevalent Cases}}_{it}}{{\text{Population}}_{it}}\times 100,000\times w_{i}\right)$$

where.


$$w_{i}=\frac{{\text{Standard Population}}_{i}}{\sum {\text{Standard population}}_{i}}$$

and *i* = age group, *t* = calendar year, Population is the age-specific female population, and *w_i_* represents the corresponding WHO standard population weight.

Standard population of people age-group data was taken from the WHO official report for standard population numbers from 2000 to 2025 (28).

Prevalence was defined as the number of women alive in calendar year *t* with a recorded cervical cancer diagnosis in the registry up to that year, divided by the corresponding female population. Because diagnosis history in the registry was available from 2014 onward, these estimates represent observed limited-duration prevalence rather than complete lifetime prevalence. Accordingly, the effective look-back period increased from 1 year in 2014 to 10 years in 2023. Thus, only the 2023 estimate approximates a full 10-year observed prevalence.

Temporal trends in ASIR, ASMR, and ASDR were visualized as annual age-standardized rates. For ASDR, 95% uncertainty intervals (UIs) were obtained using non-parametric bootstrap resampling. Per-patient DALY, YLL, and YLD estimates by stage group were summarized in tables and displayed using bar charts to describe severity at diagnosis.

Disability-adjusted life years (DALYs) were calculated at the individual level for incident cervical cancer cases as the sum of years of life lost (YLL) and years lived with disability (YLD). YLL was calculated for women who died during follow-up and was defined as the remaining life expectancy at the rounded age at death, based on the 2021 GBD life table. YLD was estimated using an incidence-based phase approach with GBD cancer disability weights and attributed to the year of diagnosis. For annual ASDR estimation, individual DALYs were aggregated by diagnosis year and age group, divided by the corresponding age-specific female population denominator, and age-standardized using the WHO standard population. Specifically, time after diagnosis was divided into a primary therapy phase of 1.0 year (disability weight [DW] = 0.2875), a middle phase extending from the end of primary therapy until censoring or the terminal phase, and a terminal phase corresponding to the last 0.25 years before target-cause death (DW = 0.53956). The middle phase was assigned a metastatic DW of 0.45136 for Stage IV/M1 disease and a controlled-phase DW of 0.04901 otherwise. For each year, we derived point estimates of ASDR and corresponding 95% UIs from a non-parametric bootstrap of the analytical dataset with 500 replicates; UIs were defined as the 2.5th and 97.5th percentiles of the bootstrap distribution. As a sensitivity analysis, DALYs were additionally recalculated using a prevalence-based approach. In this approach, YLDs were allocated to the calendar years in which disability was lived rather than exclusively to the year of diagnosis. For each patient, follow-up time after diagnosis was split into calendar-year intervals and assigned to phase-specific cancer health states using the same GBD disability weights as in the main analysis. YLLs were attributed to the calendar year in which death occurred. Annual prevalence-based DALYs were calculated as the sum of calendar-year YLDs and death-year YLLs, and age-standardized DALY rates were computed using the WHO standard population. These results are presented in the **Supplementary Materials**.

To characterize disease severity in the presentation, we aggregated individual DALY, YLL, and YLD values by stage at diagnosis. TNM stage was grouped into four categories: “Early (I–II)”, “Advanced (III)”, “Advanced (IV/M1)”, and “Unknown”. For each group, we calculated the mean DALY, YLL, and YLD per incident case and the corresponding number of cases.

In the survival analysis, the start date was defined as the day of the diagnosis (if the date of diagnosis was missing, the date of first admission was used), and the follow-up was until 11 October 2024 (day of registry download), or until the date of death. The primary survival endpoint was all-cause mortality.

### 2.5 Statistical analysis

Data cleaning and statistical analysis were performed using Stata 18.5 MP version (StataCorp, STATA Statistical Software: Release 18. College Station, TX: StataCorp LLC; 2023), and selected burden-of-disease, survival, and forecasting analyses were conducted in Python 3.10 (64 bit) (pandas, lifelines, statsmodels, pmdarima, xgboost, and matplotlib libraries). Descriptive statistics summarized patient demographics and clinical features. Continuous variables were reported as means with standard deviations, and categorical variables were reported as frequencies and percentages.

#### 2.5.1 Survival analysis and Cox regression

Overall survival by stage at diagnosis was estimated using Kaplan–Meier methods. To identify factors associated with mortality, the Cox proportional hazards (PH) regression models were fitted. Candidate covariates were selected based on clinical relevance and statistical significance (*p* < 0.05) in univariable models. A multivariable Cox model was built using stepwise backward elimination guided by the Akaike Information Criterion (AIC). Multicollinearity was assessed using variance inflation factors (VIFs), and no variable exceeded commonly accepted thresholds. Results from univariable and multivariable models are reported as hazard ratios (HRs) and adjusted hazard ratios (AHRs) with 95% confidence intervals (CIs). The PH assumption was evaluated using Schoenfeld-type residuals (Python lifelines check_assumptions function). Model performance and goodness of fit were further evaluated using likelihood-based statistics (AIC) and model discrimination was assessed using Harrell’s concordance index (C-index). The overall level of statistical significance was set at α< 0.05.

#### 2.5.2 Restricted mean survival time

To provide an absolute measure of survival differences, restricted mean survival time (RMST) up to 1, 2, 3, 4, and 5 years of follow-up was estimated. RMST was computed as the area under the Kaplan–Meier survival curve from time 0 to restriction time (τ). We calculated 5-year RMST for key categorical variables (TNM stage and treatment group) and for binary comorbidities (cardiovascular diseases, metabolic/endocrine disorders, genitourinary/reproductive conditions, hematologic/immune diseases, infectious/parasitic diseases, mental/behavioral disorders, symptoms/signs/abnormal findings, and non-malignant neoplasms). For categorical variables, pre-specified reference categories were “Stage I” and surgery-based treatment; for comorbidities, the reference level was absence of the condition (0 vs 1).

For each exposure, the difference in 5-year RMST relative to the reference group (ΔRMST, expressed in days) and the RMST ratio (RMST exposed/RMST reference) were estimated. Non-parametric bootstrap resampling with 1,000 iterations was used to derive 95% CIs and two-sided *p*-values for RMST differences. For categorical variables, RMST difference curves were calculated across restriction times of τ = 1, 2, 3, 4, and 5 years. These trajectories were summarized for disease stage and treatment variables, together with curves for all comorbidities, and presented in a multi-panel figure. All RMST analyses were implemented in Python using the lifelines Kaplan–Meier estimator and custom integration routines.

#### 2.5.3 Time-series forecasting of incidence and mortality

Time-series forecasting was performed to estimate future cervical cancer incidence rates in Kazakhstan over a 10-year horizon (2024–2033), using both monthly and yearly aggregated data.

Incidence data were aggregated from individual-level diagnosis dates (diag_date) for January 2014–December 2023. Forecasting models were evaluated using a 1-year holdout design: observations from 2014 to 2022 were used for training, and monthly incidence in 2023 served as the test set.

Four models were compared: a seasonal naïve benchmark (value from the same month in the previous year, *t−*12), Prophet with yearly seasonality, seasonal ARIMA with annual periodicity (*m* = 12) and orders selected via auto_arima, and an ARIMA–LSTM hybrid in which an LSTM was trained on ARIMA residuals and combined with the ARIMA forecast. To account for potential pandemic-related disruption and reporting changes, a binary intervention regressor was included (March 2020–December 2021 = 1; otherwise = 0) in Prophet and as an exogenous covariate in ARIMA. Predictive performance was summarized on the 2023 holdout using mean absolute error (MAE), root mean squared error (RMSE), mean absolute percentage error (MAPE), symmetric mean absolute percentage error (sMAPE), weighted absolute percentage error (WAPE), coefficient of determination (*R*²), and mean error (bias). The best-performing model was refit to the full 2014–2023 series and used to generate 10-year projections for 2024–2033. All time-series models and visualizations were implemented in Python.

All analyses are reproducible from scripted workflows in Stata and Python. Graphical outputs and summary statistics (including RMST estimates and time-series model metrics) were exported to external files for reporting and **Supplementary Material**.

### 2.6 Ethical considerations

The study was approved by the Institutional Research Ethics Committee of Nazarbayev University (protocol code: NU-IREC 651/24112022 and date of approval: 28 November 2022). Because of the retrospective nature of the study, the exemption from informed consent was granted.

## 3 Results

**Figure 1:**
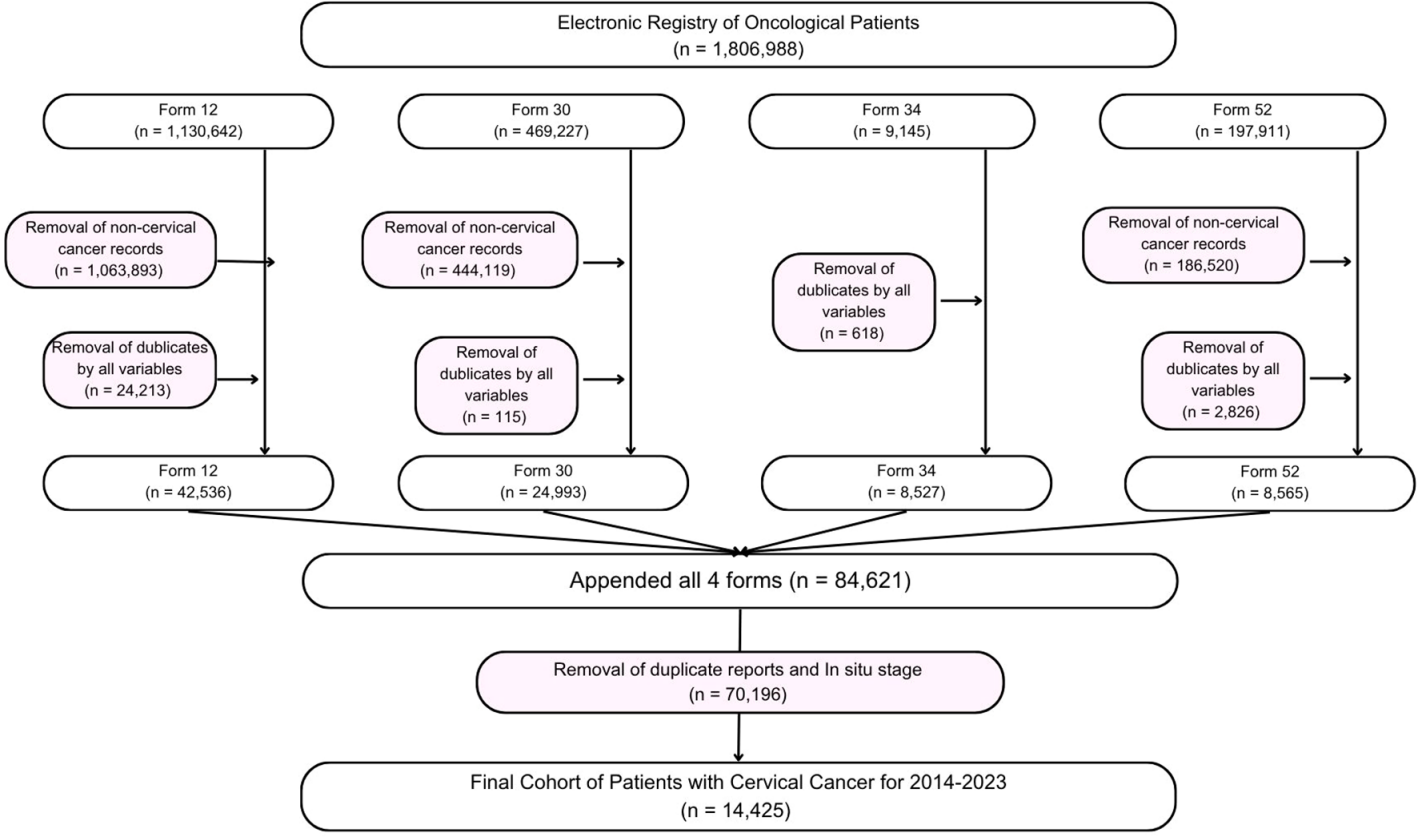
Data collection and cleaning process.

**Figure 1** shows the data cleaning and data collection process. Overall, 1,806,988 patient records were obtained, and 14,425 were used for further analysis.

### 3.1 Sociodemographic data description

The population’s mean age was approximately 52.1 years old for both alive and deceased cohorts (**Table 1**). The highest incidence was observed in the 50- to 54-year-old age group, having a bell-shaped trend. BMI characteristic was missing in 35.6% of all cases, and the highest occurrence of cervical cancer was among healthy weight patients (23.2%). Almost two-thirds (64.5%) of the population were from urban areas. It shows the lower determination rate in rural areas (35.5%). As it was expected from the highest prevalence among older ages, 26.6% of people were retired, followed by working people (24.6%), housewives (18.4%), and unemployed (17.9%).

**Table 1:** Demographic characterization of the study cohort (2014–2023).

Factor	Total	Alive*	Deceased*
*N*	14,425	9,733	4,692
Age, mean (SD)	52.1 (12.4)	51.2 (12.0)	54.1 (12.9)
Year			
2014	843 (5.8%)	528 (5.4%)	315 (6.7%)
2015	947 (6.6%)	599 (6.2%)	348 (7.4%)
2016	1,103 (7.6%)	706 (7.3%)	397 (8.5%)
2017	1,425 (9.9%)	958 (9.8%)	467 (10.0%)
2018	1,418 (9.8%)	942 (9.7%)	476 (10.1%)
2019	1,499 (10.4%)	891 (9.2%)	608 (13.0%)
2020	1,642 (11.4%)	994 (10.2%)	648 (13.8%)
2021	1,830 (12.7%)	1,209 (12.4%)	621 (13.2%)
2022	1,885 (13.1%)	1,408 (14.5%)	477 (10.2%)
2023	1,833 (12.7%)	1,498 (15.4%)	335 (7.1%)
Age groups			
0–14	1 (<1%)	0 (0.0%)	1 (<1%)
15–19	4 (<1%)	4 (<1%)	0 (0.0%)
20–24	35 (0.2%)	28 (0.3%)	7 (0.1%)
25–29	284 (2.0%)	213 (2.2%)	71 (1.5%)
30–34	850 (5.9%)	647 (6.6%)	203 (4.3%)
35–39	1,420 (9.8%)	1,024 (10.5%)	396 (8.4%)
40–44	1,881 (13.0%)	1,345 (13.8%)	536 (11.4%)
45–49	1,970 (13.7%)	1,313 (13.5%)	657 (14.0%)
50–54	2,060 (14.3%)	1,378 (14.2%)	682 (14.5%)
55–59	1,983 (13.7%)	1,339 (13.8%)	644 (13.7%)
60–64	1,624 (11.3%)	1,107 (11.4%)	517 (11.0%)
65–69	1,170 (8.1%)	735 (7.6%)	435 (9.3%)
70–74	623 (4.3%)	370 (3.8%)	253 (5.4%)
75–79	294 (2.0%)	151 (1.6%)	143 (3.0%)
>80	226 (1.6%)	79 (0.8%)	147 (3.1%)
BMI category			
Underweight	396 (2.7%)	176 (1.8%)	220 (4.7%)
Healthy weight	3,352 (23.2%)	2,039 (20.9%)	1,313 (28.0%)
Overweight	2,857 (19.8%)	1,944 (20.0%)	913 (19.5%)
Obesity	2,352 (16.3%)	1,557 (16.0%)	795 (16.9%)
Severe obesity	331 (2.3%)	186 (1.9%)	145 (3.1%)
Missing	5,137 (35.6%)	3,831 (39.4%)	1,306 (27.8%)
Nationality			
Kazakh	8,404 (58.3%)	5,969 (61.3%)	2,435 (51.9%)
Russian	3,923 (27.2%)	2,423 (24.9%)	1,500 (32.0%)
Others	2,098 (14.5%)	1,341 (13.8%)	757 (16.1%)
Residence			
Rural	5,116 (35.5%)	3,293 (33.8%)	1,823 (38.9%)
Urban	9,309 (64.5%)	6,440 (66.2%)	2,869 (61.1%)
Social status			
Retired	3,833 (26.6%)	2,285 (23.5%)	1,548 (33.0%)
Employed (including self-employed)	3,546 (24.6%)	2,605 (26.8%)	941 (20.1%)
Housewife	2,659 (18.4%)	1,820 (18.7%)	839 (17.9%)
Unemployed	2,589 (17.9%)	1,712 (17.6%)	877 (18.7%)
Other	507 (3.5%)	390 (4.0%)	117 (2.5%)
Disabled	375 (2.6%)	199 (2.0%)	176 (3.8%)
Military	51 (0.4%)	42 (0.4%)	9 (0.2%)
Special status (homeless/convicted)	17 (0.1%)	9 (0.1%)	8 (0.2%)
Education level			
Secondary education	711 (4.9%)	555 (5.7%)	156 (3.3%)
Higher education	124 (0.9%)	107 (1.1%)	17 (0.4%)
Children	6 (<1%)	4 (<1%)	2 (<1%)
Primary education	4 (<1%)	3 (<1%)	1 (<1%)
Student/Child	3 (<1%)	2 (<1%)	1 (<1%)

*By the time of registry download (11 October 2024).

**Figure 2:**
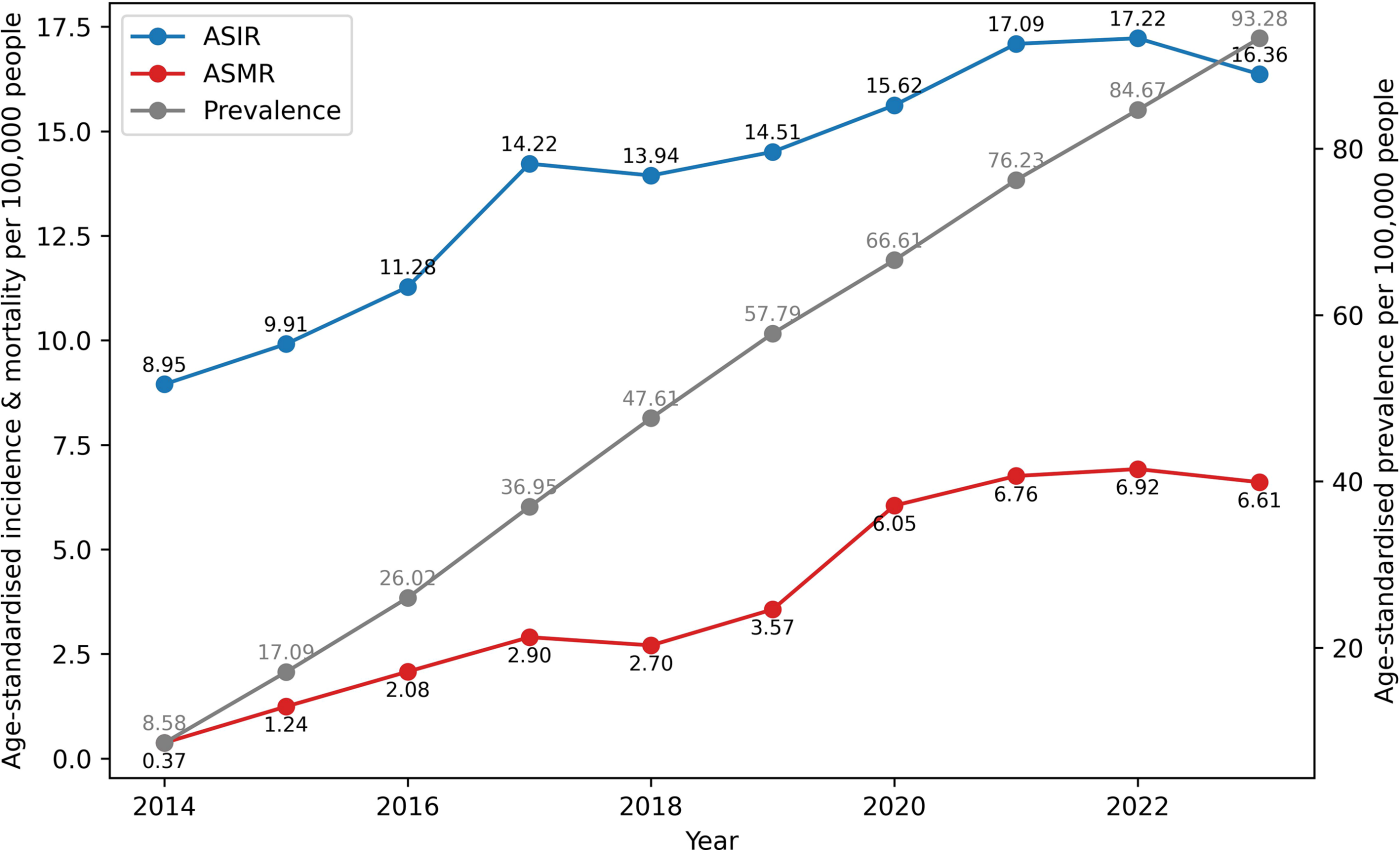
Age-standardized incidence and mortality rates, and prevalence of cervical cancer in the study cohort for the 10-year period.

**Figure 2** shows the age-standardized incidence, mortality, and prevalence of cervical cancer during the 10-year period (2014–2023). The trends are clearly seen as prevalence is constantly rising (from 8.95 to 16.36 cases per 100,000 people). ASMR showed an abrupt increase in 2020 (from 3.57 cases to 6.05 per 100,000 people). The same sharp increase is seen in ASIR (from 15.62 in 2020 to 17.09 in 2021 per 100,000 women). It can be because of the COVID-19 quarantine period restrictions and the sharp incline once restrictions were softened. From 2022 year, both ASIR and ASMR showed a declining trend, reaching 16.36 cases per 100,000 and 6.61 deaths per 100,000 women, respectively.

### 3.2 Disability-adjusted life years

**Figure 3:**
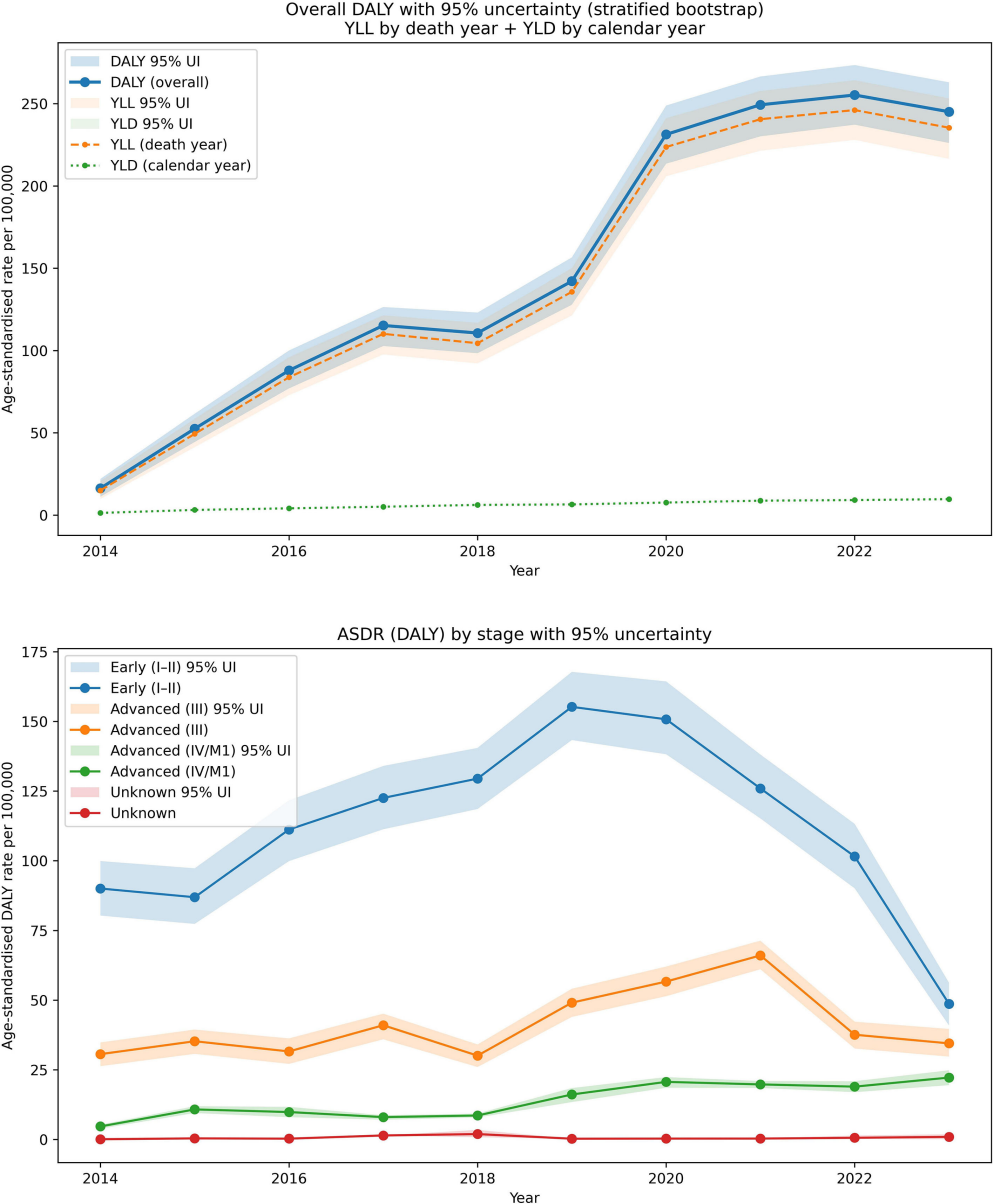
Age-standardized disability-adjusted life years. Top: overall DALY; bottom: DALY by stage at registration.

To place the observed incidence and mortality patterns in a broader population-health context, we translated them into DALYs (**Figure 3**; **Supplementary Tables 2**, **3**). This metric combines YLL due to cervical cancer deaths with years lived with cancer-related disability among incident cases.

The age-standardized DALY rate from cervical cancer increased steeply over the study period, from 16.32 per 100,000 women in 2014 (95% UI 11.18–22.00) to a peak of 255.24 per 100,000 in 2022 (95% UI 237.22–273.45), before a slight decline to 245.09 per 100,000 in 2023 (95% UI 226.10–262.96). This rise was driven almost entirely by YLL, whereas YLD remained comparatively small, rising from 1.34 per 100,000 to 9.71 per 100,000 over the study period.

When stratified by stage, DALYs were dominated by women diagnosed with early-stage disease (I–II). The ASDR-DALY for early stages increased from 90.04 per 100,000 in 2014 to a maximum of 125.96 per 100,000 in 2021, then declined to 48.63 per 100,000 in 2023. In contrast, Stage III contributed a smaller but rising burden, from 30.59 per 100,000 in 2014 to 66.03 per 100,000 in 2021, remaining elevated thereafter (34.04 per 100,000 in 2023). It is important to note that Stage III showed a fluctuating pattern, rising and declining year after year. The burden from Stage IV also increased over time, from 4.65 per 100,000 in 2014 to 22.18 per 100,000 in 2023, indicating a growing contribution of metastatic disease to overall health loss. DALYs attributed to cases with unknown stage were consistently low (approximately 0.01–2 per 100,000 each year). Together, these patterns suggest that the rapid escalation in overall DALYs has been driven mainly by fatal outcomes, particularly among women diagnosed with early- and locally advanced disease, with a progressively increasing contribution from metastatic cases in recent years.

In the supplementary prevalence-based sensitivity analysis, annual DALY estimates were highly consistent with the main incidence-based results. The prevalence-based age-standardized DALY rate increased from 16.2 per 100,000 women in 2014 to 239.4 per 100,000 women in 2023, with the highest value observed in 2022 at 249.2 per 100,000 women (**Supplementary Tables 4**, **5**). Similar to the main analysis, YLL was the dominant contributor to DALYs throughout the study period, accounting for approximately 96.3% of the prevalence-based age-standardized DALY rate in 2023.

Overall, the figure demonstrates that the growing population burden is primarily caused by increasing YLL, especially in early−stage disease, while YLD contributes little. The stage−specific trends highlight a recent peak in early−stage DALY followed by a downturn, whereas advanced disease continues a modest rise, underscoring the need for interventions that prevent premature mortality across all stages.

### 3.3 Clinical data analysis

**Figure 4:**
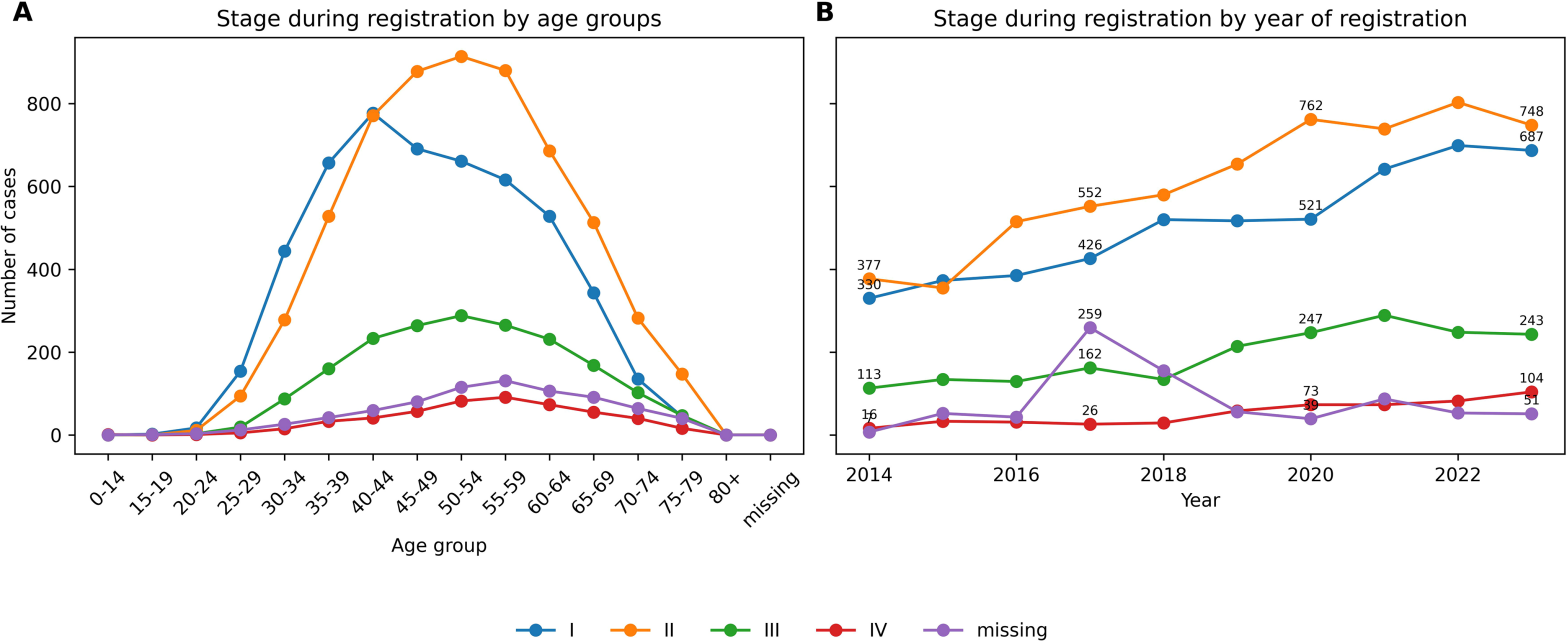
Cervical cancer staging. **(A)** Stage (by TNM classification) during registration by age groups. **(B)** Stage (by TNM classification) by registration year.

In **Table 2**, the detailed clinical profile of the population is presented. It is seen that among alive patients, almost 30.5% experience improvement in their conditions, and 23.6% experienced recovery. However, the majority of the cases (32.5%) deceased. Unfortunately, in 5.6% of live cases, stage was not determined, which affects the precision of the results. Nevertheless, most cases were registered at Stage II (42.2%), followed by Stage I (35.4%) by the TNM staging, showing a great system work in early determination of the disease. In **Figures 4A, B**, the distribution of the stages by age group and by year was shown, respectively. It is seen that the 40- to 64-year-old cohort has the highest weight among all population; however, at the same time, missing data on stage increases with age too. Based on **Figure 4B**, the increasing trend is seen in Stages I and II, justifying earlier detection of the cancer, while Stage IV and missing stay is almost unchanged. Stage III registration has an increasing trend too, but less sharply. Cervical cancer is coded under several ICD-10 codes showing the place of malignant neoplasm, namely, ICD-10 codes “C53” (cervix uteri), “C53.0” (endocervix), “C53.1” (exocervix), “C53.8” (overlapping lesion of the cervix uteri), and “C53.9” (unspecified). In the database, the highest prevalence was among the “C53.0” group (26.99%). Squamous cell carcinoma (74.9%) was the most prevalent type of cervical cancer, followed by adenocarcinoma. Here, 30% of registered cases missed information on the histological type of the cancer.

**Table 2:** Clinical characterization of the study cohort.

Factor	Total	Alive*	Deceased*
*N*	14,425	9,733	4,692
Treatment outcome			
Unchanged	580 (4.0%)	580 (6.0%)	0 (0.0%)
Recovery	3,410 (23.6%)	3,410 (35.0%)	0 (0.0%)
Transferred to a 24-h hospital	4 (<1%)	4 (<1%)	0 (0.0%)
Death	4,692 (32.5%)	0 (0.0%)	4,692 (100.0%)
Improvement	4,401 (30.5%)	4,401 (45.2%)	0 (0.0%)
Deterioration	1 (<1%)	1 (<1%)	0 (0.0%)
Missing	1,337 (9.3%)	1,337 (13.7%)	0 (0.0%)
Stage (TNM)			
I	5,100 (35.4%)	4,558 (46.8%)	542 (11.6%)
II	6,085 (42.2%)	3,546 (36.4%)	2,539 (54.1%)
III	1,913 (13.3%)	757 (7.8%)	1,156 (24.6%)
IV	525 (3.6%)	98 (1.0%)	427 (9.1%)
missing	802 (5.6%)	774 (8.0%)	28 (0.6%)
Stage (FIGO)			
I	5,248 (36.4%)	4,668 (48.0%)	580 (12.4%)
II	6,337 (43.9%)	3,675 (37.8%)	2,662 (56.7%)
III	1,597 (11.1%)	558 (5.7%)	1,039 (22.1%)
IV	441 (3.1%)	58 (0.6%)	383 (8.2%)
missing	802 (5.6%)	774 (8.0%)	28 (0.6%)
ICD-10 code			
C53	1,219 (8.5%)	990 (10.2%)	229 (4.9%)
C53.0	4,961 (34.4%)	3,501 (36.0%)	1,460 (31.1%)
C53.1	3,478 (24.1%)	2,494 (25.6%)	984 (21.0%)
C53.8	3,205 (22.2%)	1,839 (18.9%)	1,366 (29.1%)
C53.9	1,562 (10.8%)	909 (9.3%)	653 (13.9%)
Hospitalization type			
Planned hospitalization	10,675 (74.0%)	7,094 (72.9%)	3,581 (76.3%)
Emergency	239 (1.7%)	78 (0.8%)	161 (3.4%)
Missing	3,511 (24.3%)	2,561 (26.3%)	950 (20.2%)
Morphological type of tumor			
Adenocarcinoma	970 (6.7%)	528 (5.4%)	442 (9.4%)
Squamous cell carcinoma	10,807 (74.9%)	7,078 (72.7%)	3,729 (79.5%)
Other	1,184 (8.2%)	744 (7.6%)	440 (9.4%)
Missing	1,464 (10.1%)	1,383 (14.2%)	81 (1.7%)
Reason for late diagnosis			
Incomplete examination	29 (0.2%)	7 (0.1%)	22 (0.5%)
Untimely medical examination	11 (0.1%)	1 (<1%)	10 (0.2%)
Untimely referral	1,295 (9.0%)	396 (4.1%)	899 (19.2%)
Refusal of examination	7 (<1%)	3 (<1%)	4 (0.1%)
Error of other specialists	13 (0.1%)	5 (0.1%)	8 (0.2%)
Clinical error	18 (0.1%)	5 (0.1%)	13 (0.3%)
Latent course of disease	328 (2.3%)	100 (1.0%)	228 (4.9%)
Missing	12,724 (88.2%)	9,216 (94.7%)	3,508 (74.8%)
Diagnosis circumstance			
In a female/male examination room	2,186 (15.2%)	1,425 (14.6%)	761 (16.2%)
Self-referral	5,476 (38.0%)	3,074 (31.6%)	2,402 (51.2%)
Under other circumstances	640 (4.4%)	388 (4.0%)	252 (5.4%)
Under other types medical examination	3,267 (22.6%)	2,310 (23.7%)	957 (20.4%)
During screening	1,657 (11.5%)	1,370 (14.1%)	287 (6.1%)
Missing	1,199 (8.3%)	1,166 (12.0%)	33 (0.7%)
Disease status			
Local primary process	2,720 (18.9%)	1,485 (15.3%)	1,235 (26.3%)
Metastatic/systemic process	6,571 (45.6%)	4,750 (48.8%)	1,821 (38.8%)
New primary tumor	21 (0.1%)	14 (0.1%)	7 (0.1%)
Remission/NED	304 (2.1%)	91 (0.9%)	213 (4.5%)
Relapse (organ/extra organ)	58 (0.4%)	50 (0.5%)	8 (0.2%)

*By the time of registry download (11 October 2024).

From **Table 2**, it is also seen that the majority of the registrations were done by self-referral of the patients, rather than screening, emphasizing the situation with screening coverage.

The database under the current study had both staging reported in TNM and FIGO, so their similarity was compared in **Supplementary Table 1**. It is seen that Stages I and II had 96%+ compliance within two classifications, while Stages III and IV were the same, only in approximately 77% of cases, with Stage III underestimated by the FIGO, and Stage IV overestimated by the TNM classification.

### 3.4 Treatment of cervical cancer

Regarding the treatment applied for the patients, **Table 3** shows the data registered in the electronic system. Thus, it is seen that the majority of patients received radical treatment, while only a small proportion experienced a symptomatic one. More than half (55.1%) of deceased cases received radical treatment, and only 12.9% received palliative treatment. The table shows the type of treatments usually done, including chemotherapy, immunotherapy, radiation, surgery, and existing mixed types.

**Table 3:** Treatment types of cervical cancer.

Factor	Alive*	Deceased*
*N*	10,299	4,724
Treatment character		
Palliative	340 (3.5%)	605 (12.9%)
Other	16 (0.2%)	7 (0.1%)
Radical	5,475 (56.3%)	2,585 (55.1%)
Symptomatic	86 (0.9%)	193 (4.1%)
Missing	3,816 (39.2%)	1,302 (27.7%)
Treatment type		
Immunotherapy	1 (<1%)	2 (<1%)
Combined (surgery + radiation)	854 (8.8%)	145 (3.1%)
Combined (surgery + chemotherapy)	160 (1.6%)	138 (2.9%)
Combined (surgery + chemotherapy + radiation)	667 (6.9%)	516 (11.0%)
Other	6 (0.1%)	4 (0.1%)
Symptomatic	114 (1.2%)	258 (5.5%)
Chemoradiation	1,405 (14.4%)	1,183 (25.2%)
Radiation	728 (7.1%)	522 (11.0%)
Radiation method		
I-131 (systemic)	15 (0.2%)	17 (0.4%)
Brachytherapy	117 (1.2%)	82 (1.7%)
Non-traditional	808 (8.3%)	683 (14.6%)
Traditional	1,699 (17.5%)	1,017 (21.7%)
Radiation type		
Beta-therapy corpuscular	8 (0.1%)	4 (0.1%)
Gamma therapy	2,187 (22.5%)	1,688 (36.0%)
Other	5 (0.1%)	7 (0.1%)
Combined radiation therapy	104 (1.1%)	80 (1.7%)
Photon radiation therapy	1,345 (13.8%)	587 (12.5%)
Chemotherapeutic	356 (3.5%)	430 (9.1%)
Adjuvant (preventive)	322 (3.3%)	192 (4.1%)
Curative	228 (2.3%)	336 (7.2%)
Non-adjuvant	300 (3.1%)	253 (5.4%)
Independent	749 (7.7%)	748 (15.9%)
Surgical	2,018 (19.6%)	199 (4.2%)
No surgery or missing	6,644 (68.3%)	4,133 (88.1%)
Pelvic evisceration	10 (0.1%)	14 (0.3%)
Radical surgery (Wertheim/trachelectomy)	1,495 (15.4%)	275 (5.9%)
Other gynecological surgery	1,506 (15.5%)	236 (5.0%)
Diagnostic/minor gynecological	59 (0.6%)	27 (0.6%)
Non-gynecological/other	19 (0.2%)	7 (0.1%)

*By the time of registry download (11 October 2024).

### 3.5 Survival analysis

**Figure 5:**
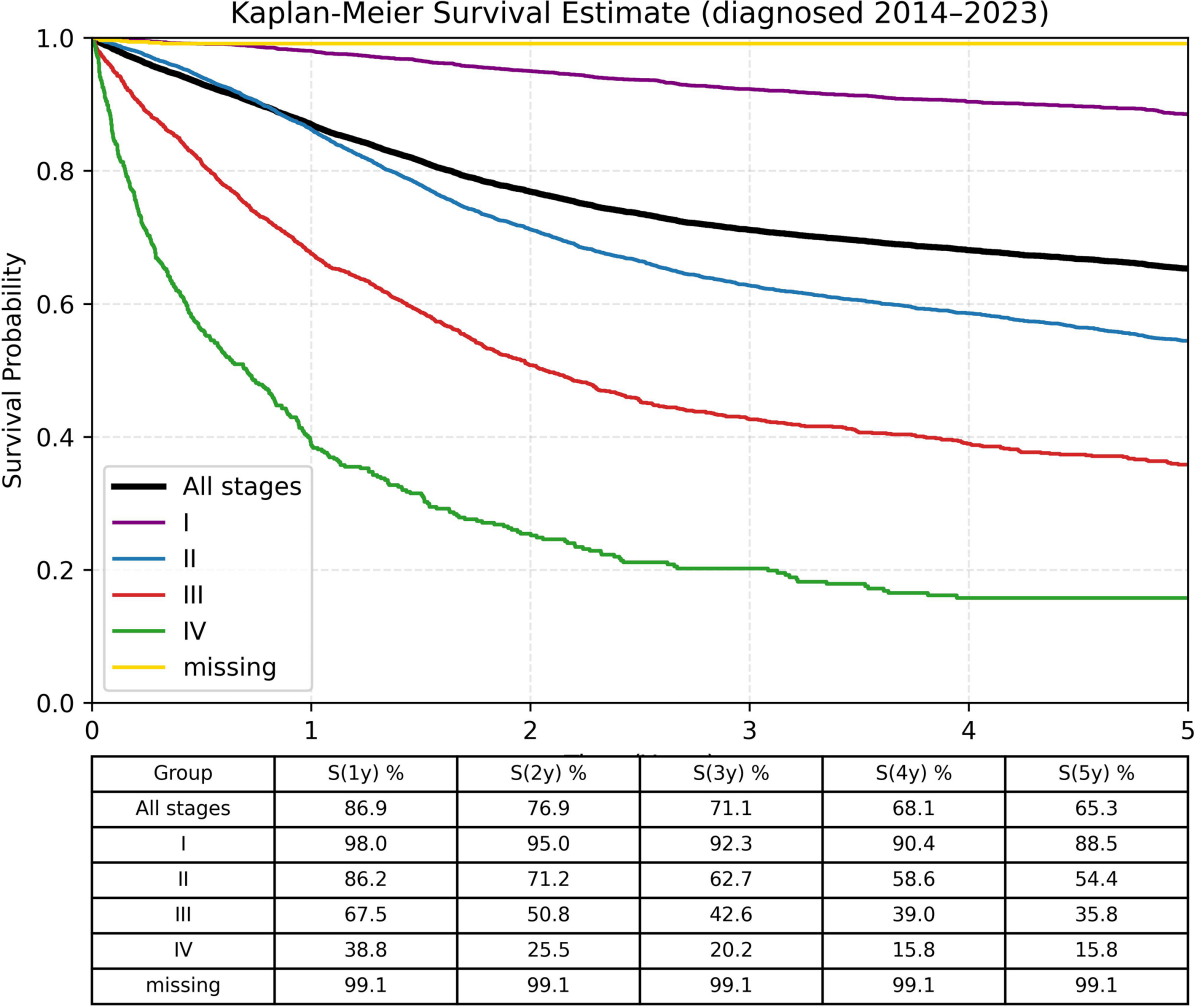
Kaplan–Meier curve by stage (based on the whole period data).

Based on the Kaplan–Meier survival analysis, the general trends are clearly seen (**Figure 5**). The label missing was preserved to see where exactly the curve went. Thus, 5-year survival for cervical cancer is equal to 65.3% in total, with greater variance in between stages (from 88.5% in Stage I, dropping to 15.8% in Stage IV). The higher the stage, the lower the survival rate was observed. The missing stage was equal to almost 1 for the whole period, showing a higher likelihood of a lower stage being diagnosed in those patients.

### 3.6 Cox regression and RMST analysis

In **Table 4**, the crude HR and AHR for the variables were calculated. The Cox regression showed that each additional decade of age increased the adjusted hazard by 22% (AHR = 1.219, *p* < 0.001) despite a non−significant crude estimate. Area of residence also affected the results, showing lower hazard in patients from urban area, compared to rural area (AHR = 0.928, *p* = 0.014). Emergency admission conferred a markedly higher risk than planned hospitalization (AHR = 1.690, *p* < 0.001). The advanced disease stage showed a steep hazard escalation, with Stage IV versus Stage I reaching an adjusted HR of 13.415 (*p* < 0.001). Treatment modality was strongly associated with outcome: chemotherapy (AHR =1.949, *p* < 0.001) and radiation (AHR =1.914, *p* < 0.001) markedly increased risk compared with surgery (reference), while combined surgery + radiation was not protective (AHR = 1.1032, *p* = 0.777). Immunotherapy was associated with a higher hazard (AHR = 5.150, *p* = 0.021); however, this subgroup included the smallest number of patients, and therefore, the estimate should be interpreted cautiously because of limited precision and potential instability. Among comorbidities, cardiovascular disease was lower than 1 (AHR = 0.680, *p* < 0.001), while mental/behavioral disorders (AHR = 1.6236, *p* < 0.001) and abnormal symptom signs (AHR = 1.315, *p* < 0.001), as well as genitourinary and hematologic immune diseases, raised hazard.

**Table 4:** Crude and adjusted hazard ratio predictors.

Variable	Crude hazard ratio			Adjusted hazard ratio		
	HR	95% CI	*P*-value	AHR	95% CI	*P*-value
Age (10-year estimate)	1.177	[1.150, 1.205]	<0.001	1.219	[1.187, 1.252]	<0.001
Residence						
Rural	Ref					
Urban	0.834	[0.786, 0.885]	<0.001	0.928	[0.874, 0.985]	0.014
Hospitalization type						
Planned	Ref					
Emergency	2.731	[2.332, 3.199]	<0.001	1.690	[1.431, 1.995]	<0.001
missing	0.688	[0.639, 0.740]	<0.001	1.333	[1.176, 1.510]	<0.001
Stage (TNM)						
I	Ref					
II	4.854	[4.423, 5.327]	<0.001	3.601	[3.253, 3.987]	<0.001
III	9.107	[8.220, 10.091]	<0.001	6.143	[5.501, 6.860]	<0.001
IV	21.022	[18.498, 23.890]	<0.001	13.415	[11.710, 15.368]	<0.001
missing	0.081	[0.040, 0.163]	<0.001	0.059	[0.030, 0.120]	<0.001
Treatment						
Surgery	Ref					
Chemotherapy	6.213	[5.240, 7.365]	<0.001	1.949	[1.631, 2.330]	<0.001
Combined (radiation+chemotherapy)	5.179	[4.448, 6.031]	<0.001	1.793	[1.525, 2.108]	<0.001
Combined (surgery+chemotherapy+radiation)	4.437	[3.760, 5.235]	<0.001	1.817	[1.529, 2.160]	<0.001
Combined (surgery+chemotherapy)	4.751	[3.818, 5.911]	<0.001	1.903	[1.521, 2.381]	<0.001
Combined (surgery+radiation)	1.355	[1.092, 1.681]	0.006	1.032	[0.831, 1.282]	0.777
Immunotherapy	8.776	[2.179, 35.345]	0.002	5.150	[1.275, 20.804]	0.021
Missing	2.656	[2.282, 3.090]	<0.001	1.464	[1.221, 1.755]	<0.001
Other	4.452	[1.654, 11.982]	0.003	1.394	[0.517, 3.762]	0.511
Radiation	4.876	[4.133, 5.752]	<0.001	1.914	[1.610, 2.276]	<0.001
Symptomatic	11.248	[9.332, 13.558]	<0.001	3.279	[2.694, 3.990]	<0.001
Comorbidities						
Cardiovascular diseases	0.762	[0.718, 0.809]	<0.001	0.680	[0.637, 0.726]	<0.001
Metabolic endocrine disorders	0.728	[0.667, 0.794]	<0.001	0.849	[0.775, 0.929]	<0.001
Genitourinary reproductive	1.499	[1.415, 1.588]	<0.001	1.402	[1.322, 1.487]	<0.001
Hematologic immune diseases	1.901	[1.761, 2.051]	<0.001	1.578	[1.459, 1.706]	<0.001
Infectious parasitic	0.758	[0.693, 0.830]	<0.001	0.837	[0.763, 0.917]	<0.001
Mental behavioral	1.873	[1.671, 2.100]	<0.001	1.636	[1.457, 1.838]	<0.001
Abnormal symptom signs	1.955	[1.721, 2.220]	<0.001	1.315	[1.154, 1.497]	<0.001
Neoplasms non-malignant	0.576	[0.504, 0.658]	<0.001	0.717	[0.627, 0.820]	<0.001

**Figure 6:**
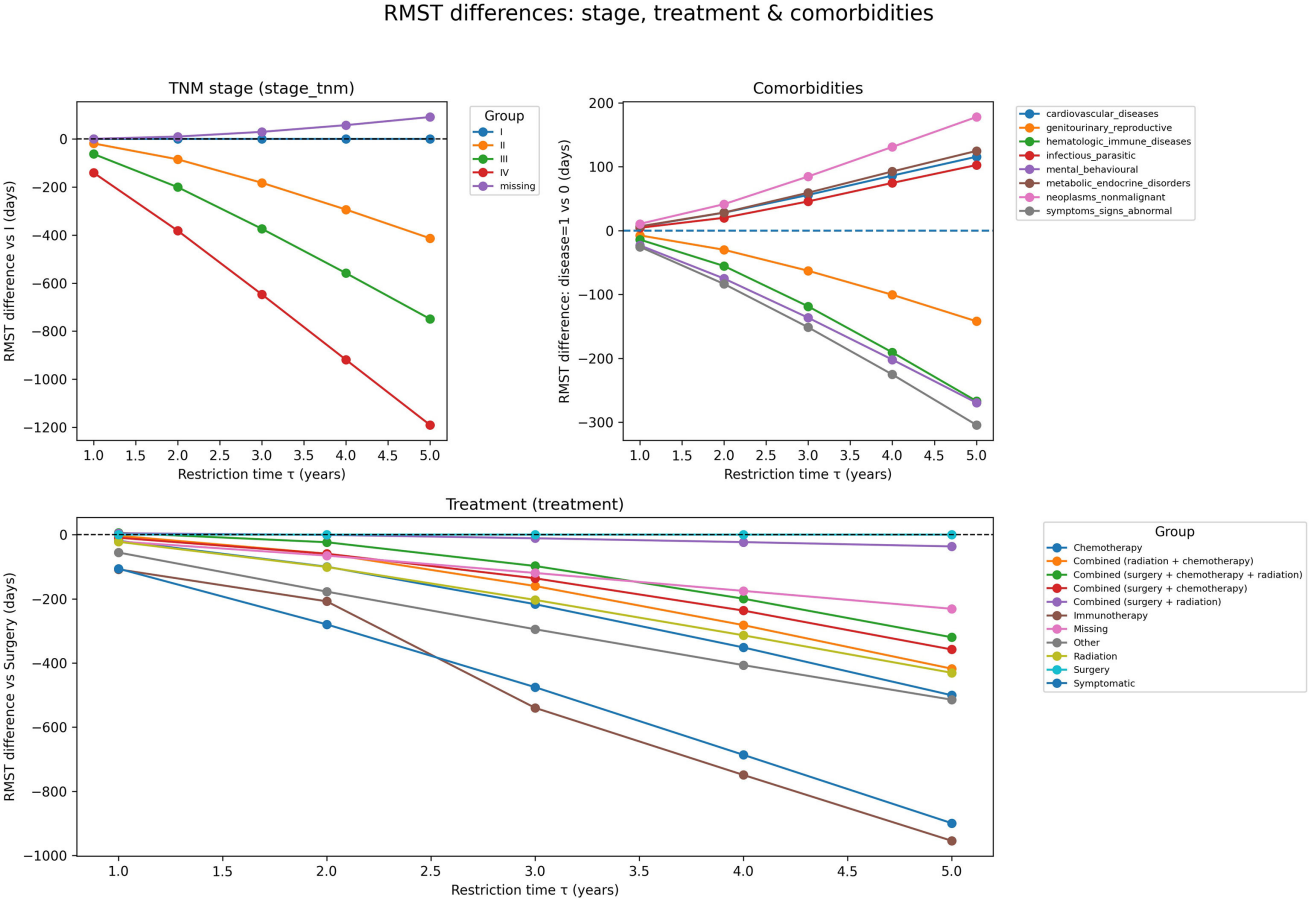
RMST analysis of non-PH variables: stage, comorbidities, and treatment type.

All variables used in the Cox regression were checked for the PH using Schoenfeld-type residuals. Because the PH assumption was not fully met for stage, comorbidities, and treatment variables (global tests *p* < 0.001), RMST was used as a complementary and more interpretable measure, as it does not assume that effects remain constant over time. Thus, HRs for stage and treatment were interpreted as average effects over follow-up rather than strictly constant over time. To provide a more interpretable and assumption-free summary of absolute survival differences, RMST differences and ratios up to 5 years were reported (29). In **Figure 6**, the top left figure shows how the difference in days changed from year 1 to year 5, compared to Stage I, taken as a reference. From the RMST analysis, it is seen that the HR differs from year to year for the stage variable (**Supplementary Table 6**). For example, patients with Stage III disease have approximately 82.6% of the average survival time of Stage I patients in the first year (≈16.7% shorter survival on average, ≈ 62 lost days). A similar increasing hazard pattern is seen for each stage category. In the comorbidity figure, results align with the trend seen in the Cox regression; the ratio did not change much throughout the 5-year period. For the “Treatment” variable, based on the ratio, a great increase in hazard in almost all treatment groups in comparison to the group of patients who underwent surgical treatment is seen.

Overall, the RMST and Cox regression do show similar comparison trends within groups; however, for the better associations for stage, comorbidities, and treatment, RMST is the better option considering their non-PH behavior.

### 3.7 Forecasting for the next 10 years

#### 3.7.1 Model validation with 1-year-ahead forecast (2023)

Four approaches discussed in the Methods section (seasonal naïve t-12, Prophet +intervention, seasonal ARIMA +intervention, and ARIMA–LSTM hybrid+ intervention) were compared to a real scenario in 1 year ahead forecast (**Supplementary Figure 2**). The seasonal ARIMA model most closely followed the observed series, while Prophet consistently overestimated incidence. As seen from the quantitative results shown in **Supplementary Table 7**, seasonal ARIMA achieved the best overall predictive accuracy, with the lowest MAE (14.55) and RMSE (19.02), as well as the lowest percentage-based errors [MAPE (10.45%), sMAPE (9.68%), and WAPE (9.53%)]. The worst model was the Prophet model (MAE 20.20; RMSE 28.12; WAPE 13.23%), while the ARIMA–LSTM hybrid showed intermediate performance (MAE 16.09; RMSE 19.02).

#### 3.7.2 Ten-year projections (2024–2033)

**Figure 7:**
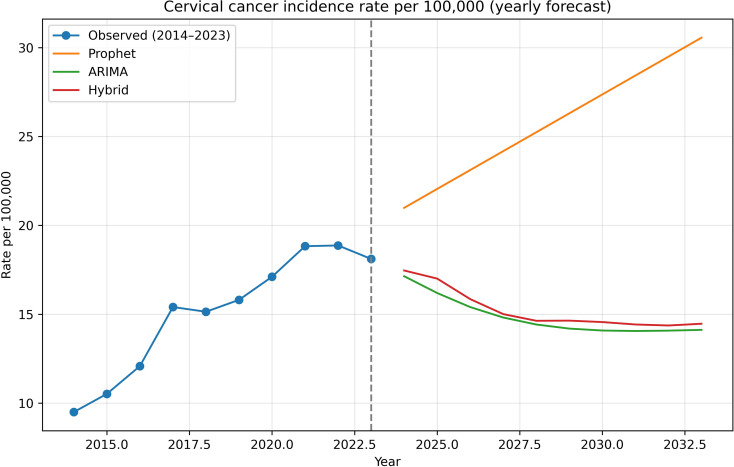
Ten-year prediction incidence forecasting till the year 2033.

**Figure 7** shows observed yearly incidence cases (2014–2023) rate per 100,000, followed by the 10-year forecast (2024–2033) with incorporated population growth. Three models were shown. Even though the best model from 1 year prediction was seasonal ARIMA, in the 10-year forecast, it aligned perfectly with the ARIMA–LSTM hybrid model, showing an increase in error once the forecasts go for longer time periods. Prophet showed the highest future burden. Overall, consistent with the 1-year forecast, seasonal ARIMA provided the most reliable basis for projection among the tested approaches, showing a slight decline in incidence rate with plateau. The monthly forecast of incidence case rate is shown in **Supplementary Figure 3**, which aligns with the yearly forecast results.

## 4 Discussion

Cervical cancer is a leading cause of cancer morbidity and mortality among women in Kazakhstan. Earlier national analyses from 1998 to 2008 reported age-standardized incidence and mortality of 14.5 and 8.0 per 100,000 women, respectively, but were based on official statistics of regional reports with no unified electronic system leading to possible under-recording (30). In the more recent reports presented by other authors for 2019, ASIR was 16.79 and ASMR was 5.56, which do not fully align with current results (8). The main reason is the difference in underlying data sources and data collection procedures. The present study used the EROP, a nationwide database established in 2012 that integrates inpatient and outpatient records from connected medical facilities. Currently, it is the most accurate database. In contrast, the previous studies were based on the Kazakhstan Cancer Registry (KCR), in which newly diagnosed cancer cases are reported by healthcare facilities to regional oncological dispensaries using a dedicated reporting form and then manually transferred into the electronic registry. This might create some overreporting or underreporting, thus decreasing the credibility of the results. However, at that time, it was the only available option for such country-scale researches. Also, different types of standardizations were used; in this study, the WHO standard population was used, while in others, European and world standards were used. This all was also shortly mentioned in the Strengths and Limitations section. As Kazakhstan rolls out a new HPV vaccination campaign, there is an urgent need for robust, individual-level evidence to characterize the true burden of disease before widespread vaccine uptake. There are meta-analyses and systematic reviews on this global burden and aimed to identify the effectiveness of campaigns; however, all studies from countries lacking pre-vaccination data were excluded (31). This study was therefore designed to provide a high-quality baseline description of cervical cancer epidemiology and survival using the national EROP database for the pre-vaccination situation in Kazakhstan.

Worldwide, cervical cancer is the fourth most common type of cancer in women, with 730,000 new cases and 373,000 deaths in 2025 (32). In this global context, Kazakhstan belongs to a group of countries with a comparatively high incidence and substantial mortality, underscoring the importance of understanding its national patterns in detail.

This study’s results reveal that the incidence rate of cervical cancer in Kazakhstan was increasing continually, reaching 17.22 cases per 100,000 women in 2022, followed by a modest decline in 2023. This level is higher than the 2022 global ASIR reported by GLOBOCAN (ASIR 14.2, ASMR 7.08 per 100,000 women) (2) and places Kazakhstan in between neighboring countries such as Kyrgyzstan (15.4 new cases per 100,000 women in 2021) and the Russian Federation (17.3 per 100,000 women), where reported incidence has also been high and mortality elevated, likely reflecting shared health-system legacies and historically absent or very limited HPV vaccination (33, 34). In contrast, age-standardized incidence and mortality in the USA and UK remain much lower (6.9 and 8.5 cases per 100,000, respectively) (35, 36), consistent with their long-standing, high-coverage HPV vaccination programs introduced in 2006 and 2008, respectively (37, 38), and comprehensive screening systems. These contrasts suggest that both the lack of established HPV vaccination, which is one of the main causes of cervical cancer (39), and suboptimal screening may contribute to the persistently high burden of cervical cancer in Kazakhstan.

At the same time, the sharp rise in incidence and mortality in Kazakhstan after 2019 coincides with the consolidation of the national electronic registry and likely reflects, at least in part, improved case ascertainment and death recording rather than a sudden surge in true risk. Thus, the observed patterns probably arise from a combination of structural prevention gaps (limited vaccination and imperfect screening) and evolving data quality.

Incidence and mortality rate show the partial picture in the national burden, while DALYs allow us to quantify both fatal and non-fatal outcomes. Kazakhstan was overwhelmingly driven by YLLs, which accounted for approximately 90%–96% of the total burden, indicating that premature death rather than long-term disability remains the dominant component. The burden increased strikingly to 245.09 in 2023, so that by the end of the study period, it exceeded the 2019 global average of 210.6 (40). Compared to the India’s 2016 DALY (223.8 per 100,000), Kazakhstan showed worse results (41). Also, the burden was much higher compared to the Eastern Mediterranean Region (DALY in 2017—86.3 per 100,000) (42). When compared to the study conducted by the GBD, Kazakhstan was in line with the average DALY in Central Asia and Central Europe, showing lower results then in low-income countries (more than 300 per 100,000), but still too high for high-income countries (less than 100 per 100,000) (43). Part of this rise may reflect improvements in case ascertainment and mortality recording as the national electronic registry matured, but it also underscores the substantial health loss attributable to cervical cancer. Stage-specific analysis for 2019 showed that early (I–II) cancers generated the largest share of DALYs, but even at these stages, most of the burden came from YLLs, indicating suboptimal survival. Overall, findings place the country in an intermediate-high burden group and highlight the urgent need to strengthen HPV vaccination, screening, and timely treatment to reduce preventable deaths.

In line with the DALY findings, survival analyses confirm that premature mortality is the main driver of burden. In Kazakhstan, the overall 5-year survival is 65.3%, ranging from 88.5% in Stage I to only 15.8% in Stage IV. This is substantially lower than the stage-specific survival reported from India (83.5%–37.1% from Stage I to IV) (44) and is almost on the same level as the pooled 5-year survival of 62.3% reported in a meta-analysis of 96 studies from Asian countries, where survival ranged from 78.9% in Turkey to 36.2% in the Philippines (45). The particularly poor outcomes in advanced stages in this study setting are consistent with the high YLL component of DALYs and likely reflect later stage at diagnosis and/or limited access to timely, guideline-based treatment.

Since the main trends are seen, it is crucial to identify risk factors associated with cervical cancer.

In multivariable Cox models, age, mode of presentation, and disease stage emerged as the strongest predictors of mortality. Each additional decade of age was associated with a 22% increase in hazard of death, emergency admission nearly doubled the hazard (AHR ≈ 1.7), and Stage IV disease carried an approximately 13-fold higher risk of death compared with Stage I. Because the PH assumption was violated for stage and treatment, we complemented Cox models with RMST analyses (46), which confirmed large survival deficits associated with non-surgical treatment. These patterns closely resemble findings from other settings. In a U.S. SEER−based nomogram of Chinese−American women, every 10 years has 59% higher hazard (HR ≈ 1.59) and Stage III increased mortality twofold, confirming the dose response between age, stage, and death (47). It is interesting to note that no IV stage was reported, meaning that early diagnosis is well developed in that country. A similar pattern is seen in a Chinese study, showing increased HR with age and stage (almost twofold both) (48). In the study conducted in Iran, an increase in BMI showed protective behavior (HR = 0.93) (49), even though in the current study, a statistical significance for the Cox regression was not received. Also, Iranian researchers report in their study that the risk of death in patients receiving at least one of the radiotherapy and chemotherapy treatments after 18 months was 7.11 times that of patients undergoing surgery (HR = 7.11; 95% CI: 1.69, 29.91) (49). It aligns with both Cox regression and RMST analysis results in this study. For chemotherapy, it was AHR = 1.949 (fifth year days difference = 500 days) and radiotherapy AHR = 1.914 (fifth year days difference = 430 days) compared to the group who received only surgery. The strong association between non-surgical therapies and poorer outcomes likely reflects confounding by indication, as chemotherapy and radiotherapy are preferentially used in more advanced cases (26).

Comorbidity analyses yielded counterintuitive inverse associations showing protective behavior. However, it is more plausibly explained by selection and detection biases: patients with chronic conditions have more frequent contact with healthcare, are more likely to undergo screening or diagnostic investigations, and may have their cause of death attributed to comorbid diseases rather than cervical cancer (50, 51). In contrast, less commonly screened conditions showed elevated hazards. It shows the importance of any kind of screening for better early detection rates.

Over the past two decades, many high-income countries have experienced substantial declines in cervical cancer incidence and mortality, largely attributable to organized screening and the introduction of HPV vaccination. For example, England’s national HPV vaccination program has been associated with an estimated 80%–90% reduction in cervical cancer risk among women vaccinated at 12–13 years of age, illustrating the transformative potential of high coverage at young ages (52). A recent publication highlighted that the potential health impact of HPV vaccines is higher than was previously forecasted, with health benefits such as reducing 15–19 cervical cancer cases, 12–14 deaths, and 243–306 DALYs for every 1,000 vaccinated 9-year-old girls, with the upper and lower limits reflecting the estimates for the nonvalent vaccine and bivalent or quadrivalent vaccines, respectively (53). Kazakhstan had earlier attempted implementing a vaccination campaign; however, it failed back in 2013–2015 (54). A new round of the campaign started in 2024, covering all 11- to 13-year-old girls. It is also interesting to note that the awareness of the HPV vaccine is higher in the rural area (58%); however, vaccination rate itself is higher in the urban area (74%) (8). Also, there is a much lower rate of “negative” discussions about the HPV vaccination campaign than it was back then, with 79% of online discussions being neutral, 14% being positive, and only 7% being negative (54), creating better opportunities for the successful establishment of the vaccination campaign. By October 2025, more than 278,200 girls had received HPV vaccination in Kazakhstan, including 170,000 girls who received the first dose (81% coverage) and 108,200 who received the second dose under the national two-dose schedule. However, according to the updated WHO 2022 recommendation, a one- or two-dose schedule is acceptable for girls aged 9–14 years, meaning that first-dose coverage is particularly important for assessing population-level progress (19, 55). The first vaccination effect in a 10- to 15-year period is expected to be seen due to the age of the vaccinating group, and with strengthened general knowledge, an increase in cervical cancer screenings can occur in the next few years. It is for this reason that forecasting models using pre-vaccination data were developed to provide a baseline for evaluating Kazakhstan’s newly relaunched HPV vaccination program. In the study by Kaviani et al., the hybrid model demonstrated better predictive performance than the ARIMA model. In contrast, our study found that ARIMA performed better for 1-year predictions, while for 10-year projections, there was little to no difference between the two models (56). It makes a valuable addition to the general knowledge and can be used for deeper analysis on which model is best for cancer incidence predictions. As for the current study, the best-performing model, seasonal-ARIMA, predicted a constant increase in the number of monthly cases from year to year. The projection was adjusted for the population growth in Kazakhstan; no vaccination effect was included, as its influence will not be seen earlier than a 10-year period. It can be assumed that national HPV vaccination can increase general knowledge among the population, leading to greater awareness and higher attendance at screening campaigns. However, it is impossible to evaluate such a secondary effect; thus, it was not included in the model. The model assumed a lower diagnosis rate during the COVID-19 years, as was described in the Methods section, but still, it should be interpreted with caution, as it does not account for possible policy shifts, new screening methods, or other disruptions. Nevertheless, projections offer a useful counterfactual: if the vaccination program and improvements in screening are successful, observed incidence should diverge favorably from these forecast trajectories over the coming decade.

### 4.1 Strengths and limitations

This study is the first nationwide study in Kazakhstan with the most precise and up-to-date data on cervical cancer epidemiology, with some clinical variables included. The present study provides a multidimensional portrait of the survival burden of cervical cancer among women in Kazakhstan, linking individual−level prognostic factors (age, BMI, disease stage, treatment modality, and comorbidities) with population−level DALY trends. The EROP database offers the most complete and rigorously curated national dataset on cervical cancer to date, encompassing all regions of the country. By integrating ASIR, ASMR, RMST differences, Cox PH estimates, age−standardized DALY, and forecasting trajectories, we can appreciate both the clinical drivers of premature death and the broader societal impact of the disease.

However, findings should also be interpreted in light of important limitations. There are no deeper details for treatment duration and series, with a lack of data on the death reason. Databases have a lot of missing data, even on very crucial variables such as stage at diagnosis, BMI, medication, social status, and education level. With those data, we can create a better profile of the target audience, but nevertheless, the current study gives a much deeper understanding of the current trends and helps to analyze the situation in the country.

## 5 Conclusion

This nationwide, registry-based analysis from Kazakhstan reveals that cervical cancer remains a major and growing public health burden, with age-standardized incidence substantially exceeding global and high-income-country averages and DALYs dominated by premature mortality. Despite a 5-year overall survival of 65.3%, outcomes in advanced stages are poor and contribute disproportionately to YLL. By combining Cox PH modeling, RMST, DALYs, and machine learning forecasts, the study results showed that age, emergency presentation, and stage at diagnosis are the key drivers of excess mortality, while inverse associations with common comorbidities likely reflect earlier detection among patients with greater healthcare contact rather than true protection.

The findings suggest that the baseline for later comparison in 10–20 years, once the HPV vaccination is fully implemented, reaches a high coverage and is completely effective. Even with an increase in screening precision, it is assumed to have much lower ASIR and ASMR compared to the forecasted numbers. Also, the study shows the risk factors and issues that should be addressed further on the national level, such as screening effectiveness and coverage, better treatment, and follow-up. All of these findings create a solid background of the situation before, to have baseline information for future comparisons, and offer valuable insights for countries with similar healthcare and vaccination contexts.

## Data Availability

The data that support the findings of this study are available from the Republican Centre for Electronic Health of the Ministry of Health of the Republic of Kazakhstan, but restrictions apply to the availability of these data, which were used under license for the current study, and so are not publicly available. Data are available from the author, Prof. AG, upon reasonable request and with permission of the Ministry of Health of the Republic of Kazakhstan.

## References

[1] Singh D Vignat J Lorenzoni V Eslahi M Ginsburg O Lauby-Secretan B . Global estimates of incidence and mortality of cervical cancer in 2020: a baseline analysis of the WHO Global Cervical Cancer Elimination Initiative. Lancet Glob Health. (2023) 11:e197–206. doi: 10.1016/s2214-109x(22)00501-0 36528031 PMC9848409

[2] WHO . Cancer today (2022). Available online at: https://gco.iarc.who.int/today/ (Accessed December 10, 2025).

[3] Kobayashi O Kamata S Okuma Y Nakajima T Ikeda Y Saito K . Carcinogenesis and epidemiology of cervical cancer: the hallmark of human papillomavirus-associated cancer. J Obstet Gynaecol Res. (2024) 50:25–30. doi: 10.1111/jog.15997 38839079

[4] Canfell K . Towards the global elimination of cervical cancer. Papillomavirus Res Amst Neth. (2019) 8:100170. doi: 10.1016/j.pvr.2019.100170 31176807 PMC6722296

[5] WHO . Global estimates of incidence and mortality of cervical cancer in 2020: a baseline analysis of the WHO Global Cervical Cancer Elimination Initiative - The Lancet Global Health (2020). Available online at: https://www.thelancet.com/journals/langlo/article/PIIS2214-109X(22)00501-0/fulltext (Accessed December 10, 2025). 10.1016/S2214-109X(22)00501-0 PMC9848409 36528031

[6] WHO . Global strategy to accelerate the elimination of cervical cancer as a public health problem (2020). Available online at: https://www.who.int/publications/i/item/9789240014107 (Accessed December 10, 2025).

[7] Igissinov N Igissinova G Telmanova Z Bilyalova Z Kulmirzayeva D Kozhakhmetova Z . New trends of cervical cancer incidence in Kazakhstan. Asian Pac J Cancer Prev APJCP. (2021) 22:1295–304. doi: 10.31557/apjcp.2021.22.4.1295 33906325 PMC8325133

[8] Zhetpisbayeva I Rommel A Kassymbekova F Semenova Y Sarmuldayeva S Giniyat A . Cervical cancer trend in the Republic of Kazakhstan and attitudes towards cervical cancer screening in urban and rural areas. Sci Rep. (2024) 14:13731. doi: 10.1038/s41598-024-64566-8 38877051 PMC11178783

[9] zur Hausen H . Papillomaviruses and cancer: from basic studies to clinical application. Nat Rev Cancer. (2002) 2:342–50. doi: 10.1038/nrc798 12044010

[10] Aimagambetova G Chan C Ukybassova T Imankulova B Balykov A Kongrtay K . Cervical cancer screening and prevention in Kazakhstan and Central Asia. J Med Screen. (2021) 28:48–50. doi: 10.1177/0969141320902482 31980007

[11] Aimagambetova G Azizan A . Epidemiology of HPV infection and HPV-related cancers in Kazakhstan: a review. Asian Pac J Cancer Prev APJCP. (2018) 19:1175–80 doi: 10.22034/APJCP.2018.19.5.1175. PMC6031825 29801397

[12] Babi A Issa T Issanov A Akilzhanova A Nurgaliyeva K Abugalieva Z . Prevalence of high-risk human papillomavirus infection among Kazakhstani women attending gynecological outpatient clinics. Int J Infect Dis IJID Off Publ Int Soc Infect Dis. (2021) 109:8–16. doi: 10.1016/j.ijid.2021.06.006 34111543

[13] Babi A Issa T Gusmanov A Akilzhanova A Issanov A Makhmetova N . Prevalence of high-risk human papilloma-virus infection and genotype distribution among Kazakhstani women with abnormal cervical cytology. Ann Med. (2023) 56:2304649. doi: 10.1080/07853890.2024.2304649 38237138 PMC10798292

[14] Balmagambetova S Tinelli A Urazayev O Sakieva K Koyshybaev A Zholmukhamedova D . HPV types distribution in general female population and in women diagnosed with cervical cancer across Western Kazakhstan. Asian Pac J Cancer Prev APJCP. (2019) 20:1089–96. doi: 10.31557/apjcp.2019.20.4.1089 31030478 PMC6948905

[15] Bekmukhambetov Y Balmagambetova S Jarkenov T Nurtayeva S Mukashev T Koyshybaev A . Distribution of high risk human papillomavirus types in Western Kazakhstan - retrospective analysis of PCR data. Asian Pac J Cancer Prev APJCP. (2016) 17:2667–72. doi: 10.7314/APJCP.2016.17.5.2667 27268648

[16] Niyazmetova L Aimagambetova G Stambekova N Abugalieva Z Seksembayeva K Ali S . Application of molecular genotyping to determine prevalence of HPV strains in Pap smears of Kazakhstan women. Int J Infect Dis IJID Off Publ Int Soc Infect Dis. (2017) 54:85–8. doi: 10.1016/j.ijid.2016.11.410 27894986

[17] Babi A Issa T Issanov A Akhanova S Udalova N Koktova S . Knowledge and attitudes of mothers toward HPV vaccination: a cross-sectional study in Kazakhstan. Womens Health Lond Engl. (2023) 19:17455057231172355. doi: 10.1177/17455057231172355 37184051 PMC10192804

[18] Satanova A Bolatbekova R Kukubassov Y Ossikbayeva S Kaidarova D . Vaccination effectiveness against human papillomavirus in Kazakhstan. Asian Pac J Cancer Prev APJCP. (2024) 25:681–8. doi: 10.31557/apjcp.2024.25.2.681 38415556 PMC11077137

[19] Ministry of Health . HPV vaccination in Kazakhstan (2025). Available online at: https://betaegov.kz/memleket/entities/dsm/press/news/details/933509 (Accessed December 10, 2025).

[20] Issa T Babi A Azizan A Alibekova R Khan S Issanov A . Factors associated with cervical cancer screening behaviour of women attending gynaecological clinics in Kazakhstan: a cross-sectional study. Womens Health Lond Engl. (2021) 17:17455065211004135. doi: 10.1177/17455065211004135 33784210 PMC8013635

[21] Kaidarova D Zhylkaidarova A Dushimova Z Bolatbekova R . Screening for cervical cancer in Kazakhstan. J Glob Oncol. (2018) 4:50s–s. doi: 10.1200/jgo.18.65600 42148471

[22] Shamsutdinova A Kulkayeva G Karashutova Z Tanabayev B Tanabayeva S Ibrayeva A . Analysis of the effectiveness and coverage of breast, cervical, and colorectal cancer screening programs in Kazakhstan for the period 2021-2023: regional disparities and coverage dynamics. Asian Pac J Cancer Prev APJCP. (2024) 25:4371–80. doi: 10.31557/apjcp.2024.25.12.4371 39733430 PMC12008358

[23] Beyembetova A Ablayeva A Akhmedullin R Abdukhakimova D Biniyazova A Gaipov A . National Electronic Oncology Registry in Kazakhstan: patient’s journey. Epidemiol Health Data Insights. (2025) 1:ehdi004. doi: 10.63946/ehdi/16385

[24] Governement . Digital journey: Kazakhstan’s healthcare. Available online at: https://betaegov.kz/memleket/entities/dsm/press/article/details/4848 (Accessed May 6, 2025).

[25] Bureau of National statistics . Agency for Strategic planning and reforms of the Republic of Kazakhstan Bureau of National statistics - Main (2025). Available online at: https://stat.gov.kz/en/ (Accessed May 6, 2025).

[26] Ministry of Healthcare, Republic of Kazakhstan . Cervical cancer. MedElement (2023). Available online at: https://diseases.medelement.com/disease/%D1%80%D0%B0%D0%BA-%D1%88%D0%B5%D0%B9%D0%BA%D0%B8-%D0%BC%D0%B0%D1%82%D0%BA%D0%B8-%D0%BA%D0%BF-%D1%80%D0%BA-2023/17756 (Accessed December 6, 2025).

[27] Boardman C . Cervical cancer staging: TNM and FIGO classifications for cervical cancer (2025). Available online at: https://emedicine.medscape.com/article/2006486-overview?form=fpf (Accessed December 6, 2025).

[28] SEER . WHO 2000-2025 standard population (2025). Available online at: https://seer.cancer.gov/stdpopulations/world.who.html (Accessed December 6, 2025).

[29] Akhmedullin R Aimyshev T Utebekov Z Kyrgyzbay G Kimadiev D Gaipov A . Epidemiology of status epilepticus in Kazakhstan: a 10-year population-based study. J Clin Med. (2025) 14:8911. doi: 10.3390/jcm14248911 41464813 PMC12733524

[30] Igissinov N Nuralina I Igissinova G Kim S Moore M Igissinov S . Epidemiological aspects of morbidity and mortality from cervical cancer in Kazakhstan. Asian Pac J Cancer Prev APJCP. (2012) 13:2345–8. doi: 10.7314/apjcp.2012.13.5.2345 22901220

[31] Drolet M Bénard É Pérez N Brisson M . Population-level impact and herd effects following the introduction of human papillomavirus vaccination programmes: updated systematic review and meta-analysis. Lancet Lond Engl. (2019) 394:497–509. doi: 10.1016/s0140-6736(19)30298-3 31255301 PMC7316527

[32] WHO . Cervical cancer (2025). Available online at: https://www.who.int/news-room/fact-sheets/detail/cervical-cancer (Accessed December 10, 2025).

[33] Muntyanu A Nechaev V Pastukhova E Logan J Rahme E Netchiporouk E . Risk factors and communities disproportionately affected by cervical cancer in the Russian Federation: a national population-based study. Lancet Reg Health Eur. (2022) 20:100454. doi: 10.1016/j.lanepe.2022.100454 35813967 PMC9256716

[34] WHO . Cervical cancer Kyrgyzstan 2021 country profile. Available online at: https://www.who.int/publications/m/item/cervical-cancer-kgz-country-profile-2021 (Accessed May 6, 2025).

[35] Gilham C Peto J . Is elimination of cervical cancer in sight in England? Prev Med. (2025) 191:108218. doi: 10.2139/ssrn.4989042 39756498

[36] SEER . Cervical cancer — Cancer stat facts (2023). Available online at: https://seer.cancer.gov/statfacts/html/cervix.html.

[37] GOV.UK . Information on the HPV vaccination from September 2023 (2023). Available online at: https://www.gov.uk/government/publications/hpv-vaccine-vaccination-guide-leaflet/information-on-the-hpv-vaccination-from-september-2023.

[38] Markowitz L Gee J Chesson H Stokley S . Ten years of human papillomavirus vaccination in the United States. Acad Pediatr. (2018) 18:S3–S10. doi: 10.1016/j.acap.2017.09.014 29502635 PMC11331487

[39] Ukybassova T Aimagambetova G Kongrtay K Kassymbek K Terzic M Makhambetova S . Correlation of HPV status with colposcopy and cervical biopsy results among non-vaccinated women: findings from a tertiary care hospital in Kazakhstan. Vaccines. (2025) 13:1151. doi: 10.3390/vaccines13111151 41295523 PMC12656960

[40] Momenimovahed Z Mazidimoradi A Maroofi P Allahqoli L Salehiniya H Alkatout I . Global, regional and national burden, incidence, and mortality of cervical cancer. Cancer Rep Hoboken NJ. (2023) 6:e1756. doi: 10.1002/cnr2.1756 36545760 PMC10026270

[41] Ramamoorthy T Kulothungan V Sathishkumar K Tomy N Mohan R Balan S . Burden of cervical cancer in India: estimates of years of life lost, years lived with disability and disability adjusted life years at national and subnational levels using the National Cancer Registry Programme data. Reprod Health. (2024) 21:111. doi: 10.1186/s12978-024-01837-7 39075548 PMC11287936

[42] Safaeian F Ghaemimood S El-Khatib Z Enayati S Mirkazemi R Reeder B . Burden of cervical cancer in the Eastern Mediterranean Region during the years 2000 and 2017: retrospective data analysis of the Global Burden of Disease Study. JMIR Public Health Surveill. (2021) 7:e22160. doi: 10.2196/22160 33978592 PMC8156112

[43] Zhao M Wu Q Hao Y Hu J Gao Y Zhou S . Global, regional, and national burden of cervical cancer for 195 countries and territories, 2007–2017: findings from the Global Burden of Disease Study 2017. BMC Women’s Health. (2021) 21:419. doi: 10.1186/s12905-021-01571-3 34922503 PMC8684284

[44] Balasubramaniam G Gaidhani R Khan A Saoba S Mahantshetty U Maheshwari A . Survival rate of cervical cancer from a study conducted in India. Indian J Med Sci. (2020) 73:1–10. doi: 10.25259/ijms_140_2020 40243708

[45] Vali M Maleki Z Nikbakht H Hassanipour S Kouhi A Nazemi S . Survival rate of cervical cancer in Asian countries: a systematic review and meta-analysis. BMC Womens Health. (2023) 23:671. doi: 10.1186/s12905-023-02829-8 38098009 PMC10722657

[46] A’Hern R . Cancer biology and survival analysis in cancer trials: restricted mean survival time analysis versus hazard ratios. Clin Oncol R Coll Radiol G B. (2018) 30:e75–80. doi: 10.1016/j.clon.2018.04.011 29776805

[47] Li L Yang Z Hou Y Chen Z . Moving beyond the Cox proportional hazards model in survival data analysis: a cervical cancer study. BMJ Open. (2020) 10:e033965. doi: 10.1136/bmjopen-2019-033965 32690495 PMC7371360

[48] Zhang Y Rong L Jiang H Mu X Zhao H . Construction and validation of nomograms for predicting overall survival and cause-specific survival in cervical cancer patients undergoing radical radiotherapy based on the SEER database. Front Med. (2025) 12. doi: 10.3389/fmed.2025.1587465 PMC12069064 40365497

[49] Hasankhani M Jahani Y Bazrafshan A Yazdizadeh A Karamoozian A . Factors affecting survival of patients with cervical cancer. Iran J Public Health. (2023) 52:2216–24. doi: 10.18502/ijph.v52i10.13860 37899927 PMC10612564

[50] Akhmedullin R Zhakhina G Issanov A Aimyshev T Sarria-Santamera A Crape B . Understanding the role of hypertension in stroke outcomes using Bayesian analysis. Sci Rep. (2025). doi: 10.1038/s41598-025-30952-z PMC12789474 41331058

[51] Rakhmankulova A Sakko Y Kerimkulov A Mamlin M Shalekenov S Zharlyganova D . Survival disparities and predictors in gastric cancer: a population-based study from Kazakhstan (2012-2023). Front Oncol. (2025) 15:1670082. doi: 10.3389/fonc.2025.1670082 41357613 PMC12678088

[52] Falcaro M Castañon A Ndlela B Checchi M Soldan K Lopez-Bernal J . The effects of the national HPV vaccination programme in England, UK, on cervical cancer and grade 3 cervical intraepithelial neoplasia incidence: a register-based observational study. Lancet Lond Engl. (2021) 398:2084–92. doi: 10.1016/s0140-6736(21)02178-4 34741816

[53] Abbas KM van Zandvoort K Brisson M Jit M . Effects of updated demography, disability weights, and cervical cancer burden on estimates of human papillomavirus vaccination impact at the global, regional, and national levels: a PRIME modelling study. Lancet Glob Health. (2020) 8:e536–44. doi: 10.1016/s2214-109x(20)30022-x 32105613 PMC7083230

[54] Tandoğan Ö Özsezer G . Traces of emotional fluctuations around the world regarding the HPV vaccine: a netnographic sentiment analysis. J Clin Med Kazakhstan. (2025) 22:47–53. doi: 10.23950/jcmk/16590

[55] KazTAG.KZ . Over 278,000 HPV vaccinations have been administered to adolescents in Kazakhstan. In: kaztag.kz Kazakhstan. (2025). Available online at: https://kaztag.kz/ru/news/svyshe-278-tys-privivok-protiv-vpch-sdelali-podrostkam-v-Kazakhstane (Accessed December 16, 2025).

[56] Kaviani E Passi K . Forecasting cancer incidence in Canada by age, sex, and region until 2026 using machine learning techniques. Algorithms. (2025) 18:265. doi: 10.3390/a18050265 30654563

