# Supplementary material for: Epidemiology, outcomes of treatment, and survival of cervical cancer in Kazakhstan: a nationwide population-based study
Source: Front Oncol. 2026 Jun 5;16:1820759. doi: 10.3389/fonc.2026.1820759 (PMC13278921; doi:10.3389/fonc.2026.1820759)
Supplement: Supplementary file 1 [file DataSheet1.docx]

**Supplementary materials**

**Supplementary Table 1. Comparison of registered cases by 2 types of stage classifications (FIGO and**

|  | Stage TNM | | | | | |
| --- | --- | --- | --- | --- | --- | --- |
| Stage FIGO |  | I | II | III | IV | missing |
|  | I | 4996 (98.0%) | 159 (2.6%) | 79 (4.1%) | 14 (2.7%) | 0 (0.0%) |
|  | II | 89 (1.7%) | 5871 (96.5%) | 338 (17.7%) | 39 (7.4%) | 0 (0.0%) |
|  | III | 13 (0.3%) | 50 (0.8%) | 1474 (77.1%) | 60 (11.4%) | 0 (0.0%) |
|  | IV | 2 (<1%) | 5 (0.1%) | 22 (1.2%) | 412 (78.5%) | 0 (0.0%) |
|  | missing | 0 (0.0%) | 0 (0.0%) | 0 (0.0%) | 0 (0.0%) | 802 (100.0%) |

**Supplementary Table 2. DALY (YLL+YLD) overall**

| year | ASDR_YLL | ASDR_YLD | ASDR_  DALY | YLL/DALY proportion | DALY_  LCL | DALY_  UCL | YLL_  LCL | YLL_  UCL | YLD_  LCL | YLD_  UCL | n_  boot |
| --- | --- | --- | --- | --- | --- | --- | --- | --- | --- | --- | --- |
| 2014 | 14.979 | 1.342 | 16.321 | 91.78% | 11.184 | 22.003 | 9.814 | 20.665 | 1.295 | 1.393 | 500 |
| 2015 | 49.370 | 3.147 | 52.517 | 94.01% | 44.414 | 61.608 | 41.289 | 58.451 | 3.077 | 3.221 | 500 |
| 2016 | 83.763 | 4.123 | 87.886 | 95.31% | 76.983 | 100.017 | 72.882 | 95.902 | 4.026 | 4.220 | 500 |
| 2017 | 110.138 | 5.104 | 115.242 | 95.57% | 102.751 | 126.426 | 97.652 | 121.371 | 4.977 | 5.216 | 500 |
| 2018 | 104.440 | 6.205 | 110.645 | 94.39% | 98.373 | 123.051 | 92.166 | 116.800 | 6.084 | 6.325 | 500 |
| 2019 | 135.631 | 6.497 | 142.128 | 95.43% | 127.832 | 156.572 | 121.319 | 150.035 | 6.362 | 6.608 | 500 |
| 2020 | 223.667 | 7.637 | 231.304 | 96.70% | 213.510 | 248.834 | 205.927 | 241.161 | 7.495 | 7.781 | 500 |
| 2021 | 240.539 | 8.775 | 249.314 | 96.48% | 230.130 | 266.488 | 221.381 | 257.664 | 8.617 | 8.914 | 500 |
| 2022 | 246.105 | 9.139 | 255.244 | 96.42% | 237.219 | 273.453 | 228.033 | 264.217 | 8.995 | 9.298 | 500 |
| 2023 | 235.383 | 9.710 | 245.094 | 96.04% | 226.101 | 262.959 | 216.449 | 253.163 | 9.567 | 9.855 | 500 |

**Supplementary Table 3. DALY by stage**

| Year | Stage Group | ASDR_DALY | ASDR_YLL | ASDR_YLD |
| --- | --- | --- | --- | --- |
| 2014 | Advanced (III) | 30.590 | 29.985 | 0.605 |
|  | Advanced (IV/M1) | 4.645 | 4.396 | 0.249 |
|  | Early (I-II) | 90.040 | 85.101 | 4.939 |
|  | Unknown | 0.050 | 0.000 | 0.050 |
| 2015 | Advanced (III) | 35.226 | 34.554 | 0.672 |
|  | Advanced (IV/M1) | 10.751 | 10.391 | 0.360 |
|  | Early (I-II) | 86.913 | 82.227 | 4.686 |
|  | Unknown | 0.351 | 0.000 | 0.351 |
| 2016 | Advanced (III) | 31.561 | 30.917 | 0.645 |
|  | Advanced (IV/M1) | 9.807 | 9.468 | 0.338 |
|  | Early (I-II) | 111.209 | 105.879 | 5.330 |
|  | Unknown | 0.255 | 0.000 | 0.255 |
| 2017 | Advanced (III) | 40.962 | 40.269 | 0.693 |
|  | Advanced (IV/M1) | 7.988 | 7.762 | 0.226 |
|  | Early (I-II) | 122.553 | 117.256 | 5.296 |
|  | Unknown | 1.402 | 0.000 | 1.402 |
| 2018 | Advanced (III) | 30.051 | 29.471 | 0.580 |
|  | Advanced (IV/M1) | 8.563 | 8.379 | 0.184 |
|  | Early (I-II) | 129.494 | 124.050 | 5.444 |
|  | Unknown | 1.903 | 1.154 | 0.749 |
| 2019 | Advanced (III) | 49.067 | 48.239 | 0.828 |
|  | Advanced (IV/M1) | 16.109 | 15.678 | 0.431 |
|  | Early (I-II) | 155.249 | 150.059 | 5.190 |
|  | Unknown | 0.238 | 0.000 | 0.238 |
| 2020 | Advanced (III) | 56.638 | 55.775 | 0.863 |
|  | Advanced (IV/M1) | 20.639 | 20.342 | 0.297 |
|  | Early (I-II) | 150.779 | 145.699 | 5.080 |
|  | Unknown | 0.276 | 0.140 | 0.136 |
| 2021 | Advanced (III) | 66.027 | 65.087 | 0.940 |
|  | Advanced (IV/M1) | 19.728 | 19.468 | 0.260 |
|  | Early (I-II) | 125.962 | 121.121 | 4.841 |
|  | Unknown | 0.288 | 0.000 | 0.288 |
| 2022 | Advanced (III) | 37.563 | 36.879 | 0.684 |
|  | Advanced (IV/M1) | 18.927 | 18.684 | 0.243 |
|  | Early (I-II) | 101.541 | 97.052 | 4.489 |
|  | Unknown | 0.562 | 0.410 | 0.152 |
| 2023 | Advanced (III) | 34.459 | 34.038 | 0.421 |
|  | Advanced (IV/M1) | 22.177 | 22.016 | 0.161 |
|  | Early (I-II) | 48.634 | 46.517 | 2.117 |
|  | Unknown | 0.904 | 0.836 | 0.068 |

**Supplementary Table 4. Prevalence-based DALY (YLL+YLD) overall**

| year | ASDR_YLL | ASDR_YLD | ASDR_  DALY | DALY_  LCL | DALY_  UCL | YLL_  LCL | YLL_  UCL | YLD_  LCL | YLD_  UCL | n_  boot |
| --- | --- | --- | --- | --- | --- | --- | --- | --- | --- | --- |
| 2014 | 14.999 | 1.214 | 16.212 | 11.475 | 21.751 | 10.267 | 20.546 | 1.167 | 1.262 | 500 |
| 2015 | 49.431 | 2.849 | 52.280 | 43.989 | 61.925 | 41.164 | 59.105 | 2.773 | 2.925 | 500 |
| 2016 | 82.350 | 3.766 | 86.116 | 74.300 | 97.917 | 70.473 | 94.153 | 3.674 | 3.853 | 500 |
| 2017 | 109.112 | 4.714 | 113.826 | 100.108 | 126.059 | 95.383 | 121.334 | 4.610 | 4.812 | 500 |
| 2018 | 102.648 | 5.743 | 108.391 | 95.245 | 121.162 | 89.548 | 115.415 | 5.629 | 5.867 | 500 |
| 2019 | 133.252 | 6.059 | 139.312 | 125.423 | 154.748 | 119.360 | 148.660 | 5.944 | 6.185 | 500 |
| 2020 | 220.624 | 7.095 | 227.719 | 209.820 | 247.486 | 202.777 | 240.303 | 6.963 | 7.236 | 500 |
| 2021 | 236.229 | 8.040 | 244.269 | 226.174 | 261.966 | 218.187 | 254.002 | 7.905 | 8.186 | 500 |
| 2022 | 240.863 | 8.360 | 249.222 | 230.165 | 270.532 | 221.839 | 262.110 | 8.227 | 8.485 | 500 |
| 2023 | 230.462 | 8.907 | 239.369 | 222.535 | 256.903 | 213.611 | 247.994 | 8.769 | 9.047 | 500 |

**Supplementary Table 5. Prevalence-based DALY by stage**

| Year | Stage Group | ASDR_DALY | ASDR_YLL | ASDR_YLD |
| --- | --- | --- | --- | --- |
| 2014 | Advanced (III) | 8.141 | 7.989 | 0.151 |
|  | Advanced (IV/M1) | 1.824 | 1.803 | 0.021 |
|  | Early (I-II) | 6.238 | 5.206 | 1.032 |
|  | Unknown | 0.010 | 0.000 | 0.010 |
| 2015 | Advanced (III) | 16.826 | 16.449 | 0.376 |
|  | Advanced (IV/M1) | 3.907 | 3.829 | 0.078 |
|  | Early (I-II) | 31.465 | 29.153 | 2.312 |
|  | Unknown | 0.082 | 0.000 | 0.082 |
| 2016 | Advanced (III) | 22.151 | 21.724 | 0.427 |
|  | Advanced (IV/M1) | 10.814 | 10.683 | 0.132 |
|  | Early (I-II) | 52.985 | 49.943 | 3.042 |
|  | Unknown | 0.166 | 0.000 | 0.166 |
| 2017 | Advanced (III) | 36.252 | 35.731 | 0.521 |
|  | Advanced (IV/M1) | 8.008 | 7.846 | 0.162 |
|  | Early (I-II) | 69.163 | 65.535 | 3.627 |
|  | Unknown | 0.404 | 0.000 | 0.404 |
| 2018 | Advanced (III) | 26.634 | 26.115 | 0.519 |
|  | Advanced (IV/M1) | 8.707 | 8.539 | 0.168 |
|  | Early (I-II) | 71.132 | 66.859 | 4.273 |
|  | Unknown | 1.919 | 1.136 | 0.783 |
| 2019 | Advanced (III) | 31.130 | 30.517 | 0.613 |
|  | Advanced (IV/M1) | 9.535 | 9.304 | 0.231 |
|  | Early (I-II) | 98.188 | 93.431 | 4.757 |
|  | Unknown | 0.459 | 0.000 | 0.459 |
| 2020 | Advanced (III) | 47.002 | 46.155 | 0.847 |
|  | Advanced (IV/M1) | 15.180 | 14.850 | 0.330 |
|  | Early (I-II) | 165.042 | 159.481 | 5.561 |
|  | Unknown | 0.495 | 0.138 | 0.357 |
| 2021 | Advanced (III) | 64.180 | 63.227 | 0.953 |
|  | Advanced (IV/M1) | 22.969 | 22.564 | 0.405 |
|  | Early (I-II) | 156.662 | 150.438 | 6.224 |
|  | Unknown | 0.459 | 0.000 | 0.459 |
| 2022 | Advanced (III) | 61.043 | 60.102 | 0.941 |
|  | Advanced (IV/M1) | 17.339 | 16.955 | 0.384 |
|  | Early (I-II) | 169.988 | 163.402 | 6.586 |
|  | Unknown | 0.852 | 0.403 | 0.448 |
| 2023 | Advanced (III) | 54.534 | 53.623 | 0.911 |
|  | Advanced (IV/M1) | 25.939 | 25.478 | 0.461 |
|  | Early (I-II) | 157.988 | 150.878 | 7.110 |
|  | Unknown | 0.907 | 0.483 | 0.424 |

**Supplementary Table 6. RMST difference in days, ratio**

| Group | Variable |  | Year 1 | Year 2 | Year 3 | Year 4 | Year 5 | CI, p-value for 5th year |
| --- | --- | --- | --- | --- | --- | --- | --- | --- |
| Stage | I | Difference in days | 0 | 0 | 0 | 0 | 0 |  |
|  |  | RMST ratio | 1 | 1 | 1 | 1 | 1 |  |
|  | II | Difference in days | -18.931 | -85.033 | -182.562 | -293.925 | -413.579 | [-432.87; -391.40]; p<0.001 |
|  |  | RMST ratio | 0.948 | 0.881 | 0.827 | 0.789 | 0.759 |  |
|  | III | Difference in days | -62.814 | -200.778 | -373.875 | -558.397 | -749.245 | [-782.42; -713.12]; p<0.001 |
|  |  | RMST ratio | 0.826 | 0.719 | 0.646 | 0.598 | 0.564 |  |
|  | IV | Difference in days | -140.679 | -381.762 | -646.725 | -918.77 | -1190.201 | [-1248.28; -1131.89]; p<0.001 |
|  |  | RMST ratio | 0.611 | 0.465 | 0.388 | 0.339 | 0.307 |  |
|  | missing | Difference in days | 0.268 | 9.57 | 29.149 | 57.075 | 90.855 | [75.01; 107.22]; p<0.001 |
|  |  | RMST ratio | 1.001 | 1.013 | 1.028 | 1.041 | 1.053 |  |
| Treatment characteristics | Chemotherapy | Difference in days | -19.112 | -99.868 | -216.649 | -351.544 | -500.566 | [-551.67; -447.09]; p<0.001 |
|  |  | RMST ratio | 0.946 | 0.857 | 0.79 | 0.742 | 0.704 |  |
|  | Combined (radiation + chemotherapy) | Difference in days | -3.413 | -59.29 | -160.036 | -281.966 | -417.764 | [-449.95; -385.15]; p<0.001 |
|  |  | RMST ratio | 0.99 | 0.915 | 0.845 | 0.793 | 0.753 |  |
|  | Combined (surgery + chemotherapy + radiation) | Difference in days | 5.791 | -23.533 | -97.599 | -199.508 | -319.822 | [-360.03; -283.09]; p<0.001 |
|  |  | RMST ratio | 1.016 | 0.966 | 0.906 | 0.854 | 0.811 |  |
|  | Combined (surgery + chemotherapy) | Difference in days | -9.448 | -59.422 | -135.967 | -236.615 | -357.871 | [-429.62; -283.12]; p<0.001 |
|  |  | RMST ratio | 0.973 | 0.915 | 0.868 | 0.827 | 0.788 |  |
|  | Combined (surgery + radiation) | Difference in days | 4.306 | -0.708 | -11.207 | -23.196 | -36.638 | [-71.02; -0.47]; p=0.042 |
|  |  | RMST ratio | 1.012 | 0.999 | 0.989 | 0.983 | 0.978 |  |
|  | Immunotherapy | Difference in days | -108.067 | -207.785 | -539.727 | -749.015 | -954.199 | [-1692.12; 138.94]; p=0.152 |
|  |  | RMST ratio | 0.694 | 0.702 | 0.478 | 0.451 | 0.436 |  |
|  | Missing | Difference in days | -21.249 | -65.166 | -119.33 | -175.253 | -231.074 | [-259.03; -203.49]; p<0.001 |
|  |  | RMST ratio | 0.94 | 0.906 | 0.884 | 0.872 | 0.863 |  |
|  | Other | Difference in days | -55.517 | -177.585 | -294.793 | -406.682 | -514.465 | [-1110.69; -3.84]; p=0.049 |
|  |  | RMST ratio | 0.843 | 0.745 | 0.715 | 0.702 | 0.696 |  |
|  | Radiation | Difference in days | -22.192 | -100.686 | -203.338 | -313.693 | -430.49 | [-475.48; -385.15]; p<0.001 |
|  |  | RMST ratio | 0.937 | 0.855 | 0.803 | 0.77 | 0.745 |  |
|  | Symptomatic | Difference in days | 0 | 0 | 0 | 0 | 0 |  |
|  |  | RMST ratio | 1 | 1 | 1 | 1 | 1 |  |
| Comorbidities | Cardiovascular diseases | Difference in days | -105.86 | -279.718 | -475.377 | -686.307 | -899.31 | [-983.59; -823.74]; p<0.001 |
|  |  | RMST ratio | 0.701 | 0.599 | 0.54 | 0.497 | 0.468 |  |
|  | Metabolic endocrine disorders | Difference in days | 6.162 | 28.109 | 55.849 | 86.128 | 115.725 | [94.40; 138.84]; p<0.001 |
|  |  | RMST ratio | 1.018 | 1.045 | 1.063 | 1.076 | 1.085 |  |
|  | Genitourinary reproductive | Difference in days | 6.701 | 28.294 | 59.317 | 92.608 | 124.808 | [97.70; 150.93]; p<0.001 |
|  |  | RMST ratio | 1.02 | 1.045 | 1.066 | 1.081 | 1.09 |  |
|  | Hematologic immune diseases | Difference in days | -7.476 | -30.058 | -62.895 | -100.519 | -142.032 | [-165.70; -121.75]; p<0.001 |
|  |  | RMST ratio | 0.978 | 0.954 | 0.933 | 0.917 | 0.904 |  |
|  | Infectious parasitic | Difference in days | -14.106 | -55.451 | -118.686 | -190.759 | -266.997 | [-302.55; -229.56]; p<0.001 |
|  |  | RMST ratio | 0.959 | 0.914 | 0.871 | 0.839 | 0.814 |  |
|  | Mental behavioural | Difference in days | 4.403 | 20.07 | 45.736 | 74.682 | 102.684 | [74.40; 132.51]; p<0.001 |
|  |  | RMST ratio | 1.013 | 1.032 | 1.051 | 1.065 | 1.074 |  |
|  | Symptoms signs abnormal | Difference in days | -23.125 | -75.089 | -136.483 | -202.089 | -269.569 | [-329.75; -207.06]; p<0.001 |
|  |  | RMST ratio | 0.932 | 0.883 | 0.851 | 0.827 | 0.81 |  |
|  | Neoplasms nonmalignant | Difference in days | -25.413 | -83.588 | -151.479 | -225.334 | -304.435 | [-373.09; -237.58]; p<0.001 |
|  |  | RMST ratio | 0.926 | 0.87 | 0.834 | 0.807 | 0.785 |  |


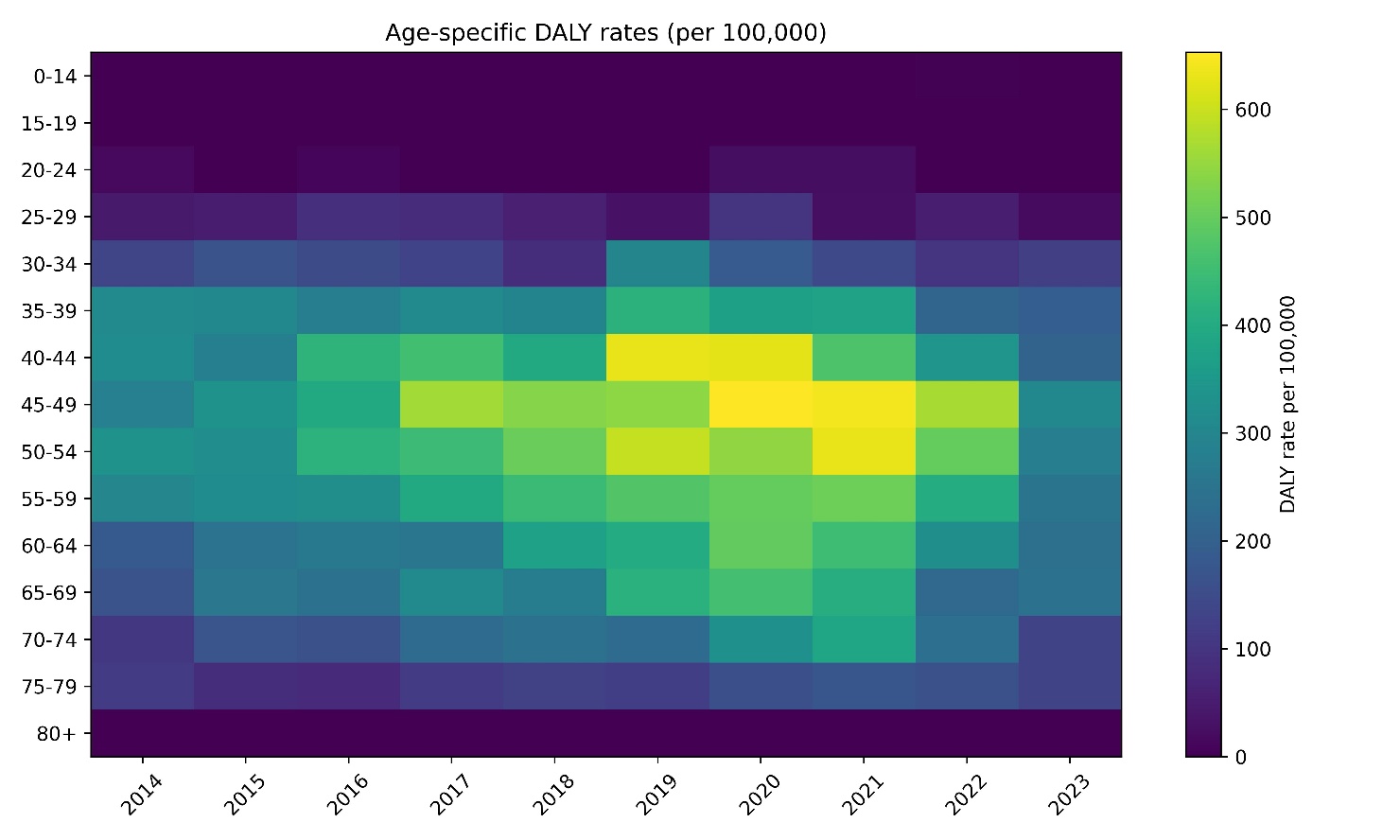


**Supplementary figure 1. Age-specific DALY rates (per 100,000)**


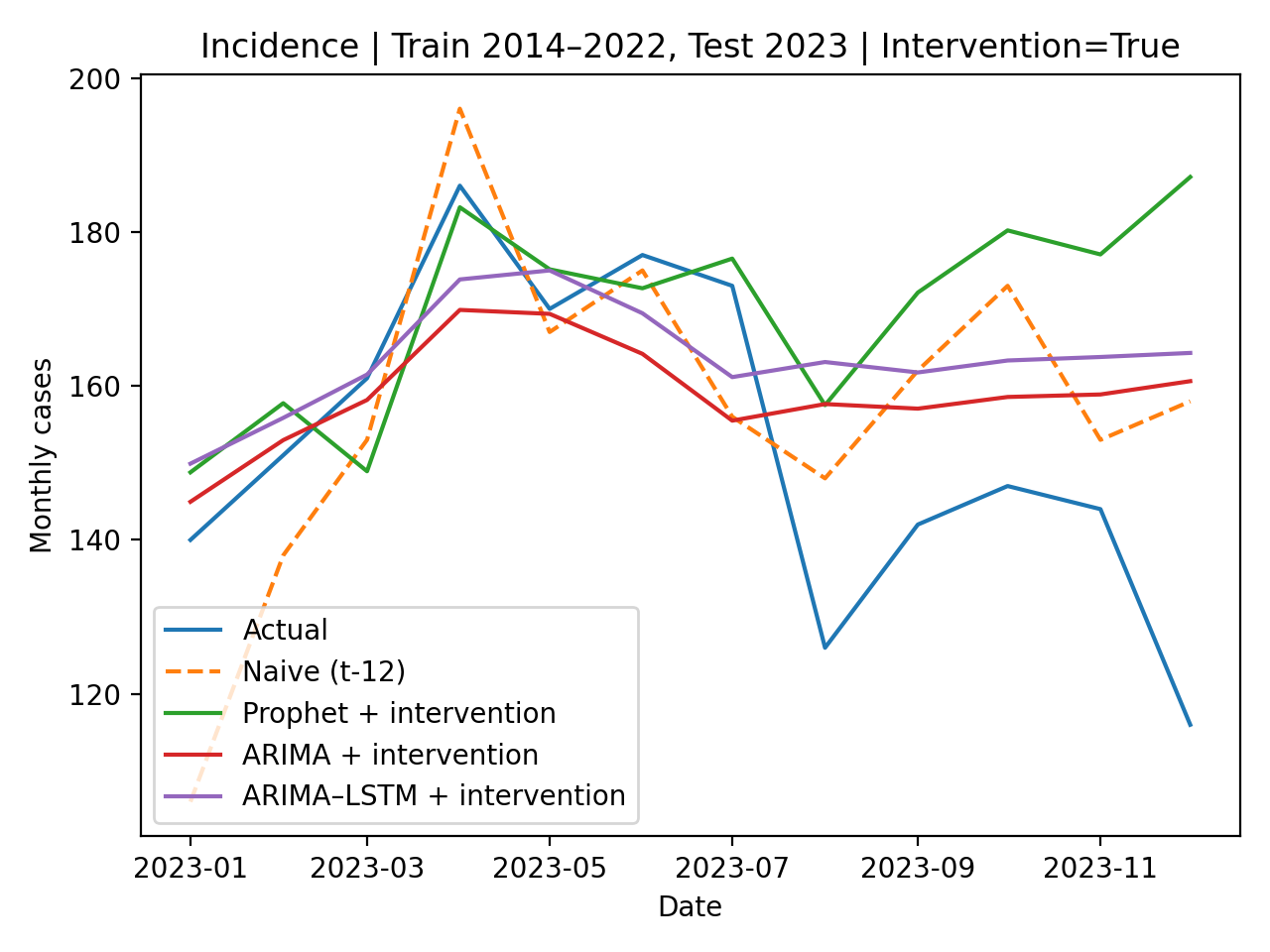


**Supplementary figure 2. One-year forecast model comparison**

**Supplementary Table 7. 2023-year forecast metrics**

| **Model** | **MAE** | **RMSE** | **R2** | **Bias (mean error)** | **MAPE % (nonzero)** | **sMAPE %** | **WAPE %** | **Total actual** | **Total predicted** | **Total abs % error** |
| --- | --- | --- | --- | --- | --- | --- | --- | --- | --- | --- |
| ARIMA | 14.552 | 19.023 | 0.118 | -6.229 | 10.45 | 9.684 | 9.527 | 1833 | 1907.752 | 4.078 |
| ARIMA-LSTM (Hybrid) | 16.085 | 20.911 | -0.065 | -10.82 | 11.749 | 10.658 | 10.53 | 1833 | 1962.838 | 7.083 |
| Naïve | 17.167 | 20.809 | -0.055 | -4.333 | 12.304 | 11.867 | 11.238 | 1833 | 1885 | 2.837 |
| Prophet | 20.204 | 28.121 | -0.926 | -17.003 | 15.029 | 13.039 | 13.227 | 1833 | 2037.037 | 11.131 |


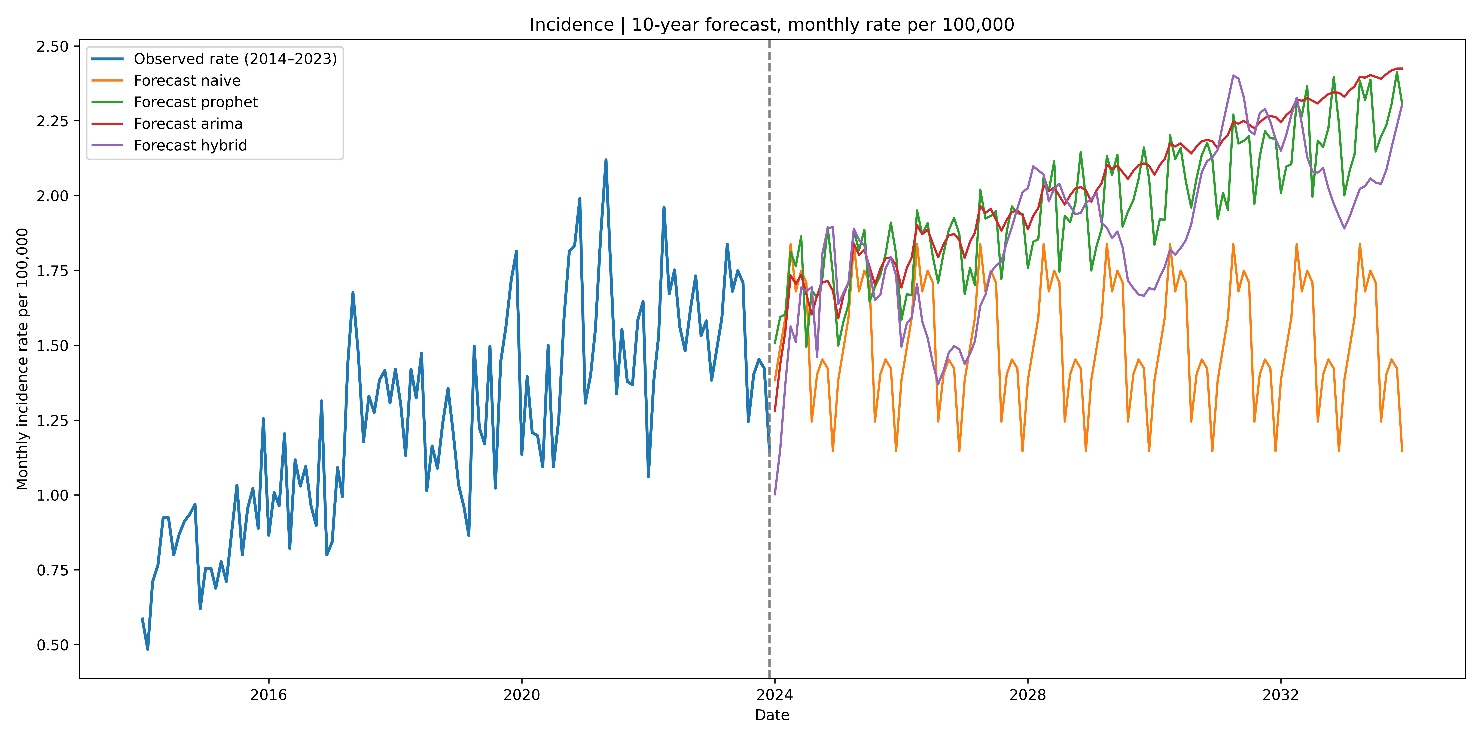


**Supplementary figure 3. Ten-year monthly forecast**

**Supplementary Table 8. Age groups distribution by stage**

| Factor | I | II | III | IV | missing |
| --- | --- | --- | --- | --- | --- |
| N | 5100 | 6085 | 1913 | 525 | 802 |
| agegroup |  |  |  |  |  |
| 0-14 | 0 (0.0%) | 0 (0.0%) | 0 (0.0%) | 1 (0.2%) | 0 (0.0%) |
| 15-19 | 2 (<1%) | 0 (0.0%) | 1 (0.1%) | 0 (0.0%) | 1 (0.1%) |
| 20-24 | 17 (0.3%) | 11 (0.2%) | 3 (0.2%) | 1 (0.2%) | 3 (0.4%) |
| 25-29 | 154 (3.0%) | 94 (1.5%) | 19 (1.0%) | 5 (1.0%) | 12 (1.5%) |
| 30-34 | 444 (8.7%) | 278 (4.6%) | 87 (4.5%) | 15 (2.9%) | 26 (3.2%) |
| 35-39 | 657 (12.9%) | 528 (8.7%) | 160 (8.4%) | 33 (6.3%) | 42 (5.2%) |
| 40-44 | 777 (15.2%) | 771 (12.7%) | 233 (12.2%) | 41 (7.8%) | 59 (7.4%) |
| 45-49 | 691 (13.5%) | 878 (14.4%) | 264 (13.8%) | 57 (10.9%) | 80 (10.0%) |
| 50-54 | 661 (13.0%) | 914 (15.0%) | 288 (15.1%) | 82 (15.6%) | 115 (14.3%) |
| 55-59 | 616 (12.1%) | 880 (14.5%) | 265 (13.9%) | 91 (17.3%) | 131 (16.3%) |
| 60-64 | 528 (10.4%) | 686 (11.3%) | 231 (12.1%) | 73 (13.9%) | 106 (13.2%) |
| 65-69 | 343 (6.7%) | 513 (8.4%) | 168 (8.8%) | 55 (10.5%) | 91 (11.3%) |
| 70-74 | 135 (2.6%) | 282 (4.6%) | 102 (5.3%) | 40 (7.6%) | 64 (8.0%) |
| 75-79 | 44 (0.9%) | 147 (2.4%) | 47 (2.5%) | 16 (3.0%) | 40 (5.0%) |
| above 80 | 31 (0.6%) | 103 (1.7%) | 45 (2.4%) | 15 (2.9%) | 32 (4.0%) |

**Supplementary Table 9. Treatment type by stage**

| Factor | I | II | III | IV | missing |
| --- | --- | --- | --- | --- | --- |
| N | 5100 | 6085 | 1913 | 525 | 802 |
| Treatment |  |  |  |  |  |
| Surgery | 1514 (29.7%) | 183 (3.0%) | 84 (4.4%) | 34 (6.5%) | 13 (1.6%) |
| Chemotherapy | 111 (2.2%) | 415 (6.8%) | 178 (9.3%) | 74 (14.1%) | 3 (0.4%) |
| Radiation | 288 (5.6%) | 708 (11.6%) | 212 (11.1%) | 37 (7.0%) | 0 (0.0%) |
| Immunotherapy | 1 (<1%) | 1 (<1%) | 0 (0.0%) | 1 (0.2%) | 0 (0.0%) |
| Combined (radiation + chemotherapy) | 177 (3.5%) | 1919 (31.5%) | 442 (23.1%) | 50 (9.5%) | 0 (0.0%) |
| Combined (surgery + chemotherapy + radiation) | 295 (5.8%) | 697 (11.5%) | 171 (8.9%) | 20 (3.8%) | 0 (0.0%) |
| Combined (surgery + chemotherapy) | 69 (1.4%) | 156 (2.6%) | 61 (3.2%) | 12 (2.3%) | 0 (0.0%) |
| Combined (surgery + radiation) | 688 (13.5%) | 243 (4.0%) | 62 (3.2%) | 6 (1.1%) | 0 (0.0%) |
| Symptomatic | 60 (1.2%) | 155 (2.5%) | 106 (5.5%) | 51 (9.7%) | 0 (0.0%) |
| Other | 3 (0.1%) | 5 (0.1%) | 1 (0.1%) | 1 (0.2%) | 0 (0.0%) |
| Missing | 1894 (37.1%) | 1603 (26.3%) | 596 (31.2%) | 239 (45.5%) | 786 (98.0%) |
